# Angio-osteogenic capacity of octacalcium phosphate co-precipitated with copper gluconate in rat calvaria critical-sized defect

**DOI:** 10.1080/14686996.2022.2035193

**Published:** 2022-02-14

**Authors:** Shinki Koyama, Ryo Hamai, Yukari Shiwaku, Tsuyoshi Kurobane, Kaori Tsuchiya, Tetsu Takahashi, Osamu Suzuki

**Affiliations:** a Division of Craniofacial Function Engineering, Tohoku University Graduate School of Dentistry, Sendai, Japan; b Division of Oral and Maxillofacial Surgery, Tohoku University Graduate School of Dentistry, Sendai, Japan; c Liaison Center for Innovative Dentistry, Tohoku University Graduate School of Dentistry, Sendai, Japan

**Keywords:** Octacalcium phosphate, copper ion, co-precipitation, osteogenesis, angiogenesis, 30 Bio-inspired and biomedical materials, 107 Glass and ceramic materials < 100 Materials

## Abstract

The objective of this study is to investigate the effects of octacalcium phosphate (OCP)-induced bone regeneration on angiogenesis regulated by the inclusion of copper ions in OCP in vitro and in vivo. Calcium (Ca)-deficient Cu-OCPs, containing 0.01 wt% Cu (low-Cu-OCP) and 0.12 wt% Cu (high-Cu-OCP), were synthesized with co7pper gluconate salt. The lattice parameters of Cu-OCPs tended to decrease slightly with Cu inclusion, as estimated by Rietveld analysis. Cu ions were released in OCP when the materials were incubated in the medium for human umbilical vein endothelial cells (HUVECs). The solubility of Cu-OCPs, estimated by the degree of supersaturation, was slightly higher than that of the original OCP. Cu-OCP tended to hydrolyze to an apatite structure while maintaining the crystal plate-like morphology when incubated with mesenchymal stem D1 cells in osteogenic media for 14 days. The specimens were characterized by selected area electron diffraction, transmission electron microscopy, and Fourier transform infrared spectroscopy. Low-Cu-OCP significantly enhanced the HUVEC capillary cross-linking density. D1 cell differentiation was inhibited with the inclusion of Cu, even at low concentrations. The composite of low-Cu-OCP with a gelatin sponge (low-Cu-OCP/Gel) significantly enhanced angiogenesis coupled with bone regeneration when implanted in a rat calvarial critical-sized defect for 4 weeks, compared with the corresponding amount of Cu-containing Gel (Cu/Gel) or OCP/Gel materials through angiography and tissue histomorphometry. These results support the proposition that angiogenesis stimulated by low-Cu-OCP is closely related with enhanced bone regeneration.

## Graphical abstract

**Figure:**
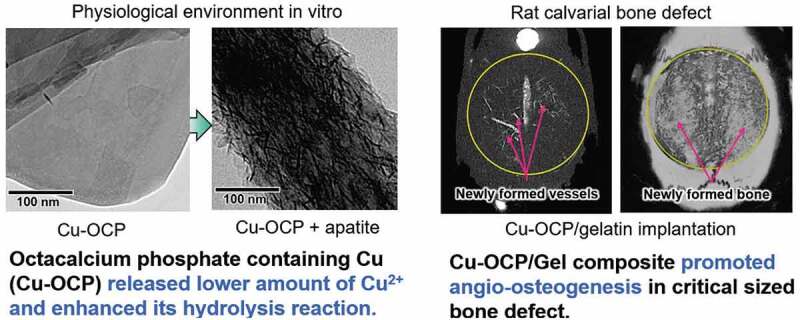

## 1. Introduction

Octacalcium phosphate (OCP) is a substance that has been advocated as a precursor for bone apatite crystals [1,2]. Its existence has been confirmed in human dentine and at the site where bone apatite crystals initiate the growth in human and rat bones [3]. OCP is non-stoichiometric, similar to hydroxyapatite (HA) [4,5], and there is a comparative report on the bone formation ability of OCP with Ca/P molar ratio values of 1.27 to 1.36 [6]. Further, the presence of Ca-deficient OCP as a cluster of HA formation has been suggested to explain the calcification of bone apatite formation [7]. The possibility of ion substitution of Mg and Zn at the cation site is predicted by first-principles calculations in Ca-deficient OCP [8], as observed in HA. Zinc-containing OCP [9], strontium-containing OCP [10] and silver-containing OCP [11] have also been synthesized. The existence of nonstoichiometric OCPs with excess HPO_4_ has been predicted [4]. OCP with approximately 40% of HPO_4_ per total P as a labile phase has been reported to be capable of exchanging HPO_4_ with the surrounding solution, which is accompanied by a reversible structural change [5]. Thus, OCP has inorganic ion-exchange capabilities and is expected to have diverse biomaterial properties.

OCP has a positive effect on bone-related cells, such as osteoblast differentiation, osteoclast formation, capillary-like tube formation of human umbilical vein endothelial cells (HUVECs), and macrophage migration in vitro [12]. The activation and accumulation of these cells have also been confirmed in vivo, corroborating that OCP promotes bone regeneration [12]. The excellent osteoconductive property of OCP was confirmed first through an onlay grafting experiment on mouse calvaria in comparison with stoichiometric HA and non-stoichiometric Ca-deficient HA materials [13]. OCP demonstrates a mechanism that promotes bone formation during progressive conversion to Ca-deficient HA [14]. Clinically oriented research has been conducted utilizing different forms of OCPs, such as OCP granules [15], blocks [16,17], and complexes with biological and synthetic polymers, such as collagen, gelatin (Gel), and poly (lactic-co-glycolic acid) (PLGA) [12]. We recently reported that the OCP/Gel complex promotes angiogenesis and bone regeneration in a rat calvarial critical-sized bone defect model compared to a Gel sponge [18]. In the aforementioned study, an adequate dose of OCP promoted HUVEC capillary-like tube formation in vitro, suggesting that OCP itself has an angiogenic potential. However, as OCP intrinsically enhances the activation of osteoblast differentiation [12,14], it remains unclear whether OCP-induced angiogenesis is involved in promoting bone regeneration.

Inorganic ions, such as Si, Zn, Ca, and Pi, have shown to be effective in activating cells, such as osteoblasts, osteoclasts, and osteocytes [19–22]. As Cu ions have various cell functions and are also involved in the expression of vascular endothelial growth factor [23], attention has been paid to the development of Cu-doped calcium phosphate that can stimulate angiogenesis [24]. Cu ions were absorbed into OCP while the crystal structure of OCP was maintained, although OCP did not have enough absorption capacity when compared with HA [25]. Based on these reports, we hypothesized that if the angiogenesis capacity of OCP is increased by incorporating Cu ions in OCP, it can be clarified whether the increase in angiogenesis itself contributes to enhancing bone regeneration induced by OCP implantation. A previous study analyzed OCP containing relatively high concentrations of Cu ions in vitro bone marrow-derived cell cultures; however, the effects on bone formation and angiogenesis in vivo has not been investigated [26].

The presence of organic molecules affects the morphology of OCP crystals, and the effects of such molecular interactions with OCP crystals have been studied for amelogenin [27], polyelectrolyte [28], collagen [29], and Gel [30]. The crystal morphology of OCP influences bone marrow-derived cell differentiation [31,32] and the bone regenerative capacity of OCP [33]. When OCP was synthesized in the presence of Cu or Zn ions, the plate-like crystals of OCP tended to change to amorphous forms depending on the ion concentration [9,26]. When angiogenesis and osteogenesis are studied by ion doping in OCP, we hypothesized that very low concentrations of Cu ion doping are required to maintain the original morphology of OCP to exclude the possible effects of the microstructure on tissue regeneration.

The present study was designed to investigate the effects of very low concentrations of Cu ion-doping in OCP on angiogenesis and its relation to bone regeneration, where the morphology of Cu-containing OCP (Cu-OCP) is similar to that of the original OCP. Special attention was directed at establishing the link between angiogenesis and bone regeneration using critical-sized rat calvarial defects.

## 2. Materials and methods

### 2.1. Preparation and characterization of OCP and Cu-OCP

OCP and Cu-OCP were prepared by a wet synthesis process [13] by mixing the calcium acetate and sodium hydrogen phosphate solutions at 65°C in the absence and presence of copper gluconate (FUJIFILM Wako Pure Chemical Co., Osaka, Japan), respectively. The degree of supersaturation with respect to OCP and HA in the mixed solution was set in the range of 4 × 10^11^ and 1 × 10^18^, respectively, during the mixing, which is calculated value at 25°C. Copper gluconate was dissolved in the reaction solution at 0.0025 and 0.025 mM to prepare the OCP containing lower (low-Cu-OCP) and higher (high-Cu-OCP) amounts of Cu, respectively. The precipitates were separated from the reaction solution and washed with pure water. Subsequently, the synthesized OCP and Cu-OCP were dried and sieved to obtain granules with diameters in the range of 300–500 μm.

The Cu content in the OCP and Cu-OCP granules was measured using inductively coupled plasma atomic emission spectroscopy (ICP-AES; iCAP6500, Thermo Fisher Scientific, Waltham, Massachusetts, USA). The contents of Ca and P in the granules were also determined using kits for Calcium E and Phosphor C tests (FUJIFILM Wako Pure Chemical Co.), respectively. The granules of OCP and Cu-OCP were dissolved in 0.1 M HCl and 0.1 M HNO_3_ solution at 1 mg/mL for the measurements using the kits and ICP-AES, respectively. 

The prepared OCP and Cu-OCP were analyzed using X-ray diffraction (XRD; Miniflex 600, Rigaku Co., Tokyo, Japan) with monochromatized Cu-Kα radiation at 40 kV and 15 mA. The X-rays were scanned from 2θ = 3° to 60° at a rate of 1.0°/min with a 0.02° step. The lattice parameters of OCP and Cu-OCP were calculated by Rietveld analysis using the PDXL2 software (version 2.8.4.0, Rigaku Co.). Silicon powder (640d, National Institute of Standards and Technology, Gaithersburg, Maryland, USA) [34] as internal standard mixed with the specimen at approximately 10 wt.% to calibrate the diffraction angle of 2θ in the patterns. The XRD patterns of specimens mixed with Si were measured in the range from 2θ = 20° to 60° for the calculation of lattice parameters. The broadening of the diffraction peaks was measured to evaluate the crystallinities of the specimens [35]. In this study, the peak corresponding to 100 diffractions, a characteristic of the lattice plane in the OCP structure, was selected. The full width at half maximum (FWHM) of the diffraction peak was measured from the patterns fitted by Rietveld analysis. The morphologies of OCP and Cu-OCP placed on 400-mesh grids (Okenshoji Co. Ltd., Tokyo, Japan) were observed using transmission electron microscopy (TEM; JEM-2100 F, JEOL Ltd., Tokyo, Japan) at an acceleration voltage of 100 kV. The crystal structures of OCP and Cu-OCP were characterized using selected area electron diffraction (SAED).

### 2.2. In vitro experiments

#### 2.2.1. HUVEC culture with OCP and Cu-OCP

Effects of Cu^2+^ in OCP on the vascularization of HUVECs in vitro were analyzed. The dry-heat sterilization of OCP, low-Cu-OCP, and high-Cu-OCP granules (1 mg) were performed at 120°C for 2 hours. The granules of OCP were mixed with low-Cu-OCP (low-Cu-OCP + OCP) and high-Cu-OCP (high-Cu-OCP + OCP) granules at a weight ratio of 1:1 to change the content of Cu in the specimens. The mixture (1.0 mg) was sterilized at 120°C for 2 hours. HUVECs were purchased from Lonza (Basel, Switzerland). Endothelial cell growth medium-2 (EGM-2) was prepared by adding supplements (EGM^TM^-2 SingleQuots™ Supplements and Growth Factors, CC-4176, Lonza Inc.) to endothelial cell basal medium−2 (EBM^TM^-2, Lonza Inc.). BD Matrigel^TM^ Matrix (100 μL, BD Bioscience, Franklin Lakes, New Jersey, USA) was coated on the bottom of a 24-well plate and then HUVECs were seeded at 1.8 × 10^5^ cells/well in 1.0 mL of EGM-2. Transwell inserts with 1.0 μm pores (FALCON® Cell Culture Insert, Corning Inc., Corning, New York, USA) were placed into each well. One milligram of the granules was placed on the inserts. The HUVECs were cultured with OCP (n = 3), low-Cu-OCP (n = 3), high-Cu-OCP (n = 3), low-Cu-OCP + OCP (n = 3), and high-Cu-OCP + OCP (n = 3) at 37°C in a humidified incubator with an atmosphere of 5% CO_2_ and 95% air for 24 hours. Furthermore, HUVECs were cultured in the presence of dissolved copper gluconate at for 24 hours (n = 3). The concentrations of copper gluconate were set to those corresponding to Cu concentrations in the media incubated with OCP, low-Cu-OCP and high-Cu-OCP granules. Cells without granules and copper gluconate were also incubated as a control group (n = 3). At 24 hours, HUVECs were observed using a Leica DMI 4000 B microscope (Leica Microsystems GmbH, Wetzlar, Germany), and five images were captured per well at 50-magnification. The total number of cross-linking points in the capillary networks of HUVECs was counted in all five captured images for each well. The number of cross-linking points was normalized by the number in the control groups to compare the capillary formation of HUVECs incubated in the presence of granules and the absence of granules while containing dissolved copper gluconate.

#### 2.2.2. Marrow-derived mesenchymal stem cell (MSC) culture with OCP and Cu-OCP

Mouse bone marrow-derived MSC line D1 cells were purchased from ATCC (Rockville, Maryland, USA), and these cells were seeded on the bottom of the 24-well plate at 2.0 × 10^4^ cells/well in 1 mL of the osteogenic medium. The medium was high-glucose Dulbecco’s Modified Eagle’s Medium (FUJIFILM Wako Pure Chemical Co.) containing 10% fetal bovine serum (ThermoFisher Scientific, Waltham, Massachusetts, USA), 1% penicillin-streptomycin mixed solution (Nacalai Tesque, Inc., Kyoto, Japan), 50 μg/mL ascorbate 2-phosphate (Sigma-Aldrich Co. LLC, St. Louis, Missouri, USA), 10 mM β-glycerophosphate (Tokyo Chemical Industry Co., Ltd., Tokyo, Japan), and 100 nM dexamethasone (Sigma-Aldrich Co. LLC). Sterilized granules of OCP (1 mg), low-Cu-OCP (1 mg), and a mixture of OCP (0.5 mg) and low-Cu-OCP (0.5 mg) were placed on the inserts in the wells. D1 cells were incubated in the presence of OCP (n = 3), low-Cu-OCP (n = 3), and low-Cu-OCP + OCP (n = 3) in 5% CO_2_ and 95% air atmosphere under humidified conditions at 37°C. Cells in the absence of granules (control group, n = 3) were also cultured. The medium was changed every 3 days. At day 7 and day 14 of incubation, the cells were washed with phosphate-buffered saline (PBS) twice and then clashed in 250 μL of 0.2% Triton X-100 solution using a sonicator. Alkaline phosphatase (ALP) activity in the incubated cells was determined using LabAssay ALP® (FUJIFILM Wako Pure Chemical Co.). Deoxyribonucleic acid (DNA) amounts in the cells were also measured using the Quant-iT™ PicoGreen® dsDNA kit (Thermo Fisher Scientific). ALP activity was normalized by the amount of DNA in the cells.

#### 2.2.3. Characterization of OCP and Cu-OCP after incubation with HUVECs and MSCs.

The granules of OCP and Cu-OCP were collected from the bottom of the inserts after incubation with HUVECs and MSCs. The collected granules were washed with ultrapure water twice and then lyophilized for two days. The morphological and crystallographic analyses of the incubated OCP and Cu-OCP were carried out using TEM (JEM-2100 F, JEOL Ltd.) and SAED, respectively. The incubated specimens were also analyzed by Fourier transform infrared (FTIR) spectroscopy (FT/IR6300; JASCO Corporation, Tokyo, Japan) with the specimens diluted in KBr.

#### 2.2.4. Measurement of ion concentrations and degree of supersaturation (DS) with respect to calcium phosphate in culture medium

The granules (1 mg) of OCP, low-Cu-OCP, and high-Cu-OCP were incubated in EGM-2 (1 mL) in the absence of HUVECs and Matrigel® at 37°C in 5% CO_2_ and 95% air for 24 hours. The supernatants were collected by centrifugation at 4400 rpm for 5 min. The concentrations of Ca^2+^ and inorganic phosphate (Pi) in the supernatants were determined using Calcium E and Phosphor C tests (FUJIFILM Wako Pure Chemical Co.), respectively. The concentration of Cu in the supernatants was also measured using ICP-AES (iCAP6500, Thermo Fisher Scientific) after the dilution of supernatants in 0.1 M HNO_3_ solution. The pH values of the supernatants were also determined using a pH electrode (9618S-10D, HORIBA Ltd., Kyoto, Japan). The DS with respect to HA, OCP, and dicalcium hydrogen phosphate dihydrate (DCPD) was calculated using Equation (1):

(1) $$DS={\left(\frac{IP}{K_{sp}}\right)}^{\frac{1}{\upsilon }}$$

where *IP* and *K_sp_* are the ionic activity products and solubility constants with respect to calcium phosphate, respectively. ν is the number of ions in calcium phosphate (ν = 9, 8 and 2, for HA, OCP and DCPD, respectively). The measured values of ion concentration and pH of the supernatants were used for the calculation of *IP* on the basis of mass balance values of Ca^2+^, Pi ions, and Mg^2+^. The calculations assumed the presence of HCO_3_^−^ and ion pairs (CaH_2_PO_4_^+^, CaHPO_4_^0^, MgHPO_4_^0^, CaHCO_3_^+^, and MgHCO_3_^+^). The ionic strength was set at 150 mM, and Na^+^ was assumed to be a background electrolyte. The concentrations of Mg^2+^ and F^−^ were assumed to be approximately zero. *K_sp_* values for HA, OCP, and DCPD were 7.36 × 10^−60^ (mol/L)^9^ [36], 2.51 × 10^−49^ (mol/L)^8^ [37], and 2.77 × 10^−7^ (mol/L)^2^ [38], respectively. DS values = 1, < 1, and > 1 indicate saturation, undersaturation, and supersaturation, respectively.

### 2.3. In vivo experiments

#### 2.3.1. Preparation and characterization of OCP/Gel, low-Cu-OCP/Gel composite, and Gel containing Cu

To evaluate the ability of OCP and Cu-OCP to induce angiogenesis and bone regeneration in vivo, composite forms of OCP/Gel and low-Cu-OCP/Gel were prepared for the stable implantation of these granules. The Cu/Gel sponge was prepared by adding copper gluconate in Gel sponge. Composites consisting of Gel sponge and OCP (OCP/Gel) or low-Cu-OCP granules (low-Cu-OCP/Gel) were prepared following the preparation method reported by Saito et al. [39]. Granules with a diameter in the range of 300–500 μm were added to a 3% porcine skin Gel (Sigma-Aldrich Co. LLC) solution at 44 wt.% to total weight of granules and gelated. Gelation of Gel was performed by homogeneously mixing the granules in the solution at 4°C. The mixture of Gel and granules was frozen at −20°C for 1 day and then lyophilized for 3 days. Gel sponges containing Cu^2+^ (Cu/Gel) were also prepared. Copper gluconate was dissolved in the Gel solution and subsequently incubated at 4°C. The Gel solution containing copper gluconate was lyophilized after freezing. The Cu content in the Gel corresponded to the amount of Cu in low-Cu-OCP, which was measured by ICP-AES, in the composites. The obtained OCP/Gel, low-Cu-OCP/Gel, and Cu/Gel were cut into disk-like shapes with a size of ϕ 9 mm × 1 mm. Finally, the dehydrothermal treatment of OCP/Gel, low-Cu-OCP/Gel, and Cu/Gel was performed under vacuum at 150°C for 24 hours to crosslink the Gel molecules.

The cross-sections of the OCP/Gel, low-Cu-OCP/Gel, and Cu/Gel were observed using scanning electron microscopy (SEM; JSM-6396LA, JEOL Ltd.) at an acceleration voltage of 10 kV before Au-Pd was coated on the surface of the specimens. The diameters of the micropores in the specimens were measured using an image analysis software (ImageJ; National Institutes of Health, Maryland, USA).

#### 2.3.2. Implantation of OCP/Gel, low-Cu-OCP/Gel, and Cu/Gel and perfusion of the angiographic agent

All procedures for animal handling and treatment were approved by the Animal Research Committee of Tohoku University (approval number 2020DnA-060). The animal experiments in this study followed the principles of standard laboratory animal care and national laws.

Wistar rats (12 weeks old, male, Japan SLC, Inc., Hamamatsu, Japan) were used to examine the vascularization and bone regeneration behaviors induced by the OCP/Gel, low-Cu-OCP/Gel, and Cu/Gel in bone defects. Anesthetization was performed via the inhalation of isoflurane by rats and subsequent injection of mixed anesthetic agents consisting of medetomidine (Nippon Zenyaku Kogyo Co., Ltd., Fukushima, Japan), midazolam (Sandoz K.K., Tokyo, Japan), and butorphanol (Meiji Seika Pharma Co., Ltd., Tokyo, Japan) into their peritoneal cavity. The dose amount of medetomidine, midazolam, and butorphanol was 0.38 µg/g, 2.0 µg/g, and 2.5 µg/g to the weight of rats, respectively. The skin and periosteum were sectioned along the bilateral line and middle of the forehead. A critical-sized defect, which does not repair spontaneously, with a diameter of 9 mm [40] was created on the line sagittal suture between the lambdoid suture and coronal suture. A trephine drill was used to create defects. The OCP/Gel (n = 5) and low-Cu-OCP/Gel (n = 5) composites were implanted into the defects. The Cu/Gel sponges (n = 5) were also implanted as control groups. After implantation, the ablated periosteum and skin were repositioned and sutured.

To perfuse the angiographic agent, the rats were anesthetized with isoflurane and mixed anesthetic agents at 2 and 4 weeks, following the same protocol described above. The heart was exteriorized by the skin section along the medial line. A vascular catheter was inserted into an incision with a diameter of 2 mm created on the left ventricular wall. All blood in the rats was removed by perfusion with 2 mL saline containing heparin (2000 units) through the inserted catheter using a peristaltic pump. Thirty milliliters of an angiographic agent (Microfil®; Flow Tech Inc. Carver, Massachusetts, USA) was perfused and subsequently hardened at 4°C for 24 hours. Finally, the calvarial tissue with the brain was collected from the animals and fixed in 10% formaldehyde solution for 3 days.

#### 2.3.3. Radiographic analysis

Radiographic analysis of the collected tissues according to the method reported by Kurobane et al. [18] was carried out to measure the volumes of newly formed calcified tissues and blood vessels in the defects. Briefly, the microfocus X-ray computed tomography (micro-CT) images of the tissues were captured using a micro-CT system (Scan XmateE090, Comscantecno Co., Ltd., Kanagawa, Japan) at 90 kV and 90 μA before and after decalcification in an ethylenediaminetetraacetic acid solution adjusted to pH 7.1 for 4 weeks. The micro-CT images were analyzed using a three-dimensional (3D) image analysis software (Amira, Thermo Fisher Scientific). A region of interest (ROI) with a diameter of 9 mm and 1.5 mm thickness was set in the defect region in the 3D micro-CT image. The position of the ROI was matched in the images of the calvarial tissues before and after demineralization. The volume of the contrasted vessels without calcified tissues in the ROI was also measured using the images after decalcification. The volume of the newly formed calcified tissue was estimated by subtracting the volume of the contrasted vessels from the measured volume of the ROI in the image before decalcification. The volume of newly formed vessels was also calculated by excluding the volume of the sagittal sinus in the ROI.

#### 2.3.4. Preparation of histological sections and histomorphometric analysis

The decalcified tissues were gradually dehydrated using an ethanol solution. The tissues were then embedded in paraffin and were cut into the cross-section of the coronal plane through the center of the defect and then sectioned at a thickness of 4 μm. The prepared thin sections were stained with hematoxylin and eosin (H&E) to evaluate the formation of new bone tissues. Images of the tissues with H&E staining were captured using a virtual slide scanner (NanoZoomer®, Hamamatsu Photonics K.K., Hamamatsu, Japan). The areas of the newly formed bone and original defects were measured by the analysis of histological images using the Image J software. The rate of the newly formed bone occupied in the defect region was calculated using Equation (2):

(2) $$\text{n-bone=}\%\left(\frac{\text{newly}\text{formed}\text{bone}\text{area}\left[{\text{mm}}^{\text{2}}\right]}{\text{original}\text{bone}\text{defect}\;\text{area}\left[{\text{mm}}^{\text{2}}\right]}\right)$$

#### 2.3.5. Immunostaining

Tissue sections, including the defect region treated with OCP/Gel and low-Cu-OCP/Gel, were deparaffinized for endomucin and vascular endothelial growth factor (VEGF) immunostaining. The antigen was activated in the tissue sections using 0.01 M citrate buffer at pH 6.0 and tris(hydroxymethyl)aminomethane/EDTA buffer at pH 9.0 for endomucin and VEGF immunostaining, respectively. The sections were incubated with primary antibodies against endocrine (dilution 1:500, #bs-5884 R, Bioss Inc., Boston, Massachusetts, USA) and VEGF (dilution 1:500, #ab1316, Abcam plc., Cambridge, UK) overnight at 4°C. The tissues were treated with 1% H_2_O_2_ in ethanol for 20 minutes to eliminate endogenous peroxidase activity after the primary antibody reaction. A secondary antibody against the peroxidase-labeled rat tissue (N-Histofine Simple Stain Rat MAX PO(M), Nichirei Bioscience Inc., Tokyo, Japan) was then reacted with the tissues. The binding secondary antibodies were detected using 3,3′-diaminobenzidine tetrahydrochloride (DAB Reagent Set, Kirkegard and Perry Laboratories Inc., Maryland, USA) which reacted with the tissues. Counterstaining was performed with hematoxylin. Immunostaining was performed using a slide scanner (NanoZoomer®, Hamamatsu Photonics K.K.). The number of endomucin- or VEGF-positive cells was counted in the images captured at 10-fold from the selected three regions (ROIs) in the defects. The total number of positive cells was normalized by the total area of the ROI in the three images.

### 2.4. Statistical analysis

Results are expressed as mean ± standard deviation (SD). Tukey-Kramer multiple comparison analysis was performed to evaluate the statistical differences among the means of multiple groups. The student's t-test was also performed to compare the means between the two groups. Statistical significance was set at p < 0.05. The Statcel 4 software (OMS Publishing Inc., Saitama, Japan) was used for all statistical analyses.

## 3. Results

### 3.1. Characterization of Cu-OCP

**Figure 1.**
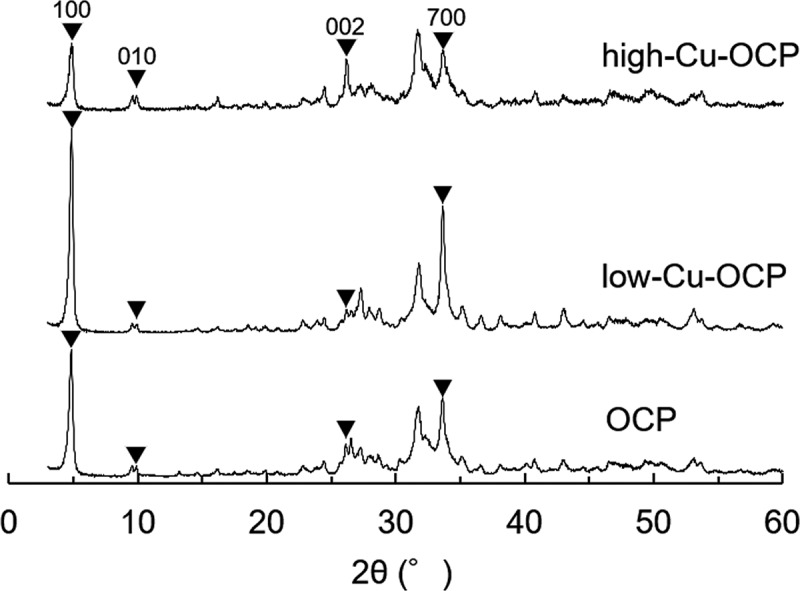
XRD patterns of OCP, low-Cu-OCP, and high-Cu-OCP.

Figure 1 shows the XRD patterns of OCP and Cu-OCP prepared in solution in the absence and presence of Cu^2+^, respectively. The diffractions corresponding to 100, 010, 002, and 700 were detected in the patterns of OCP, low-Cu-OCP, and high-Cu-OCP. Diffraction peaks corresponding to other phases, such as those of HA, were not observed in the patterns of OCP and Cu-OCP. The FWHMs of the diffraction peaks corresponding to 100 diffractions were 0.362, 0.303, and 0.388° for OCP, low-Cu-OCP, and high-Cu-OCP, respectively. These FWHM values suggest that the crystallinity of the crystal could not be markedly regulated by the presence of Cu^2+^ in the range of 0.0025 to 0.025 mM during the wet synthesis of OCP. The lattice parameters of OCP and Cu-OCP were calculated by Rietveld analysis using the XRD patterns (Table 1). The lattice parameters *a* and *b* tended to decrease with an increase in the additive amount of Cu^2+^. The lattice volumes of low-Cu-OCP and high-Cu-OCP tended to be smaller than those of the OCP prepared in this study. These calculated parameters indicate that the lattice of the OCP was contracted by increasing the amount of Cu^2+^ in the synthesis condition.

**Table 1.** Lattice parameters estimated by Rietveld analysis of OCP, low-Cu-OCP, and high-Cu-OCP

Specimens	*a* (Å)	*b* (Å)	*c* (Å)	*α* (°)	*β* (°)	*γ* (°)
OCP	19.716(5)	9.542(3)	6.856(2)	90.249(14)	92.664(14)	108.439(15)
Low-Cu-OCP	19.703(4)	9.536(2)	6.843(1)	90.135(13)	92.596(10)	108.396(10)
High-Cu-OCP	19.708(3)	9.520(1)	6.836(1)	90.031(9)	92.601(9)	108.459(8)

The contents of Ca, P, and Cu in OCP and Cu-OCP were measured by chemical analysis and ICP-AES (Table 2). The Cu content in the high-Cu-OCP was higher than that in the low-Cu-OCP. The estimation of molar ratio of Cu/(Ca+Cu) for the specimens indicated that the content of Cu as foreign ion in the present low-Cu-OCP and high-Cu-OCP were lower than that of Zn and Cu ions in OCP [9,26] reported in previous studies. The (Ca + Cu)/P molar ratio for low-Cu-OCP and high-Cu-OCP were similar to the Ca/P molar ratio for OCP (Table 2). The ratios in all specimens were lower than the stoichiometric OCP (Ca/P = 1.33). This suggests that the content of Ca-deficient sites in the crystal lattice of Cu-OCP is comparable to that in the crystal lattice of OCP.

**Table 2.** Content of Ca, P, and Cu in the specimens of OCP, low-Cu-OCP, and high-Cu-OCP and molar ratio of Cu/(Ca+Cu), Ca/P, and (Ca + Cu)/P

Specimens	Ca (wt.%)	P (wt.%)	Cu (wt.%)	Cu/(Ca+Cu)	Ca/P	(Ca+Cu)/P
OCP	32.3	19.4	–	–	1.28	–
low-Cu-OCP	31.7	19.6	0.01	0.02	–	1.25
High-Cu-OCP	31.8	19.4	0.12	0.24	–	1.27

**Figure 2.**
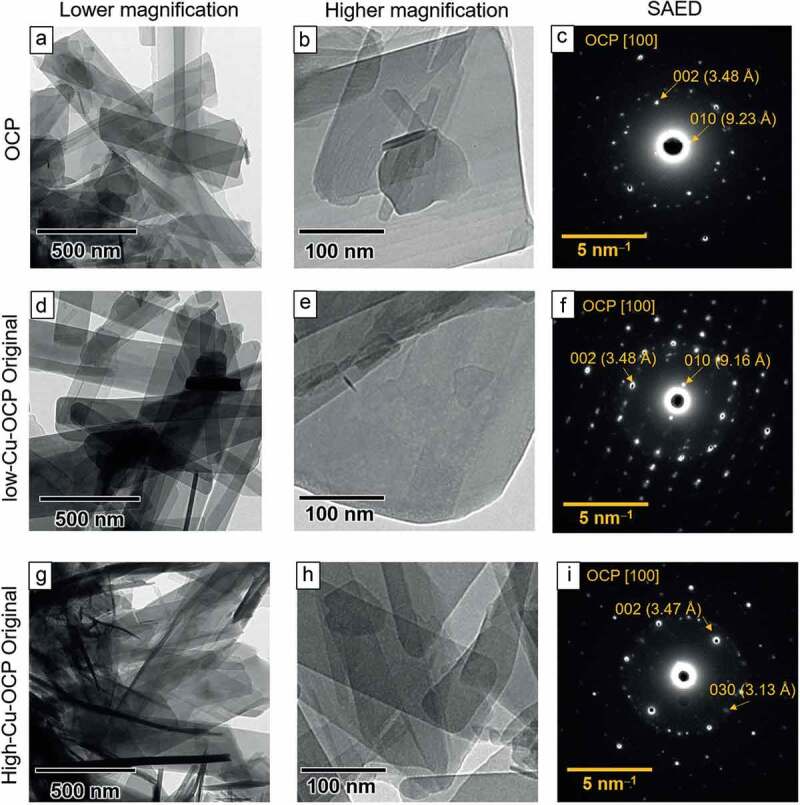
TEM images of OCP (a, b), low-Cu-OCP (d, e), and high-Cu-OCP (g, h) at low (a, d, g) and high magnifications (b, e, h). Bars in the low and high magnification images represent 500 and 100 nm, respectively. SAED patterns of OCP (c), low-Cu-OCP (f), and high-Cu-OCP (i). Bars in SAED patterns represent 5 nm^–1^.

The morphologies of the OCP and Cu-OCP were observed using TEM. A plate-like structure, a typical morphology of OCP, was observed in the bright field images of OCP (Figure 2(a)), low-Cu-OCP (Figure 2(d)), and high-Cu-OCP (Figure 2(g)) at a low magnification. A smooth surface in the absence of nanoscale depositions was observed on low-Cu-OCP (Figure 2(e)), high-Cu-OCP (Figure 2(h)), and OCP (Figure 2(b)) at a high magnification. The crystal structures of the plate-like particles of OCP and Cu-OCP were also analyzed using SAED. The diffraction spots attributed to 010 and 002 were detected at 9.23 and 3.48 Å, respectively, in the SAED patterns of OCP, which indicate that the zone axis was [100] of OCP (Figure 2(c)). The spot patterns in the images of low-Cu-OCP (Figure 2(f)) and high-Cu-OCP (Figure 2(i)) also corresponded to the zone axis of [100] of the OCP. The results of TEM and SAED analyses suggest that the plate-like particles of Cu-OCP and OCP were single crystals of OCP.

### 3.2. Change in Ca^2+^, Pi ion, and Cu concentration in the medium after the incubation of OCP and Cu-OCP

The concentrations of Ca^2+^ and Pi ions increased in EGM-2 medium incubated with OCP and Cu-OCP granules at 24 hours (Table 3)

**Table 3.** Composition and degree of supersaturation (DS) of EGM-2 medium before and after the incubations of OCP, low-Cu-OCP, and high-Cu-OCP for 24 hours. N.D. indicates the concentration was below the detection limit

Supernatants	Ca (mM)	Pi (mM)	Cu (mM)	pH	DS at 37°C		
					HA	OCP	DCPD
Original medium	1.66	0.57	N.D.	7.36	3.36 × 10^10^	1.33 × 10^2^	2.94 × 10^–1^
OCP	1.71	0.73	N.D.	7.41	5.79 × 10^10^	2.57 × 10^2^	3.81 × 10^–1^
low-Cu-OCP	1.78	0.70	1.27 × 10^–4^	7.39	1.20 × 10^11^	3.78 × 10^2^	3.89 × 10^–1^
high-Cu-OCP	1.82	0.73	1.40 × 10^–3^	7.36	1.07 × 10^11^	3.90 × 10^2^	4.09 × 10^–1^

. Although the Pi ion concentration was comparable between OCP and Cu-OCP at 24 hours, the Ca^2+^ concentration gradually increased with the increasing Cu content in the granules. The concentration of Cu in the medium incubated with high-Cu-OCP was approximately ten times higher than that of low-Cu-OCP. These Cu concentrations were in the order of micromoles per litre. The Cu concentrations in the medium before and after the incubation with OCP were below the detection limit.

The values of DS with respect to calcium phosphates were calculated based on the Ca^2+^ and Pi ion concentrations and pH measurements (Table 3). The DS with respect to DCPD was of the order of 10^–1^ in all media, which suggested that the media were undersaturated with respect to DCPD. In contrast, the DS values with respect to HA and OCP were of the order of 10^11^–10^10^ and 10^2^, respectively, which indicated that the media were supersaturated with respect to HA and OCP. The DS with respect to HA and OCP in the media incubated with OCP and Cu-OCP increased compared to the original medium. At 24 hours, the values of DS with respect to HA were in the order of 10^11^ in the media incubated with Cu-OCP, while the value was in the order of 10^10^ in the medium incubated with OCP. Furthermore, the DS with respect to HA in the low-Cu-OCP group was slightly higher than that in the high-Cu-OCP group at 24 hours.

### 3.3. Endothelial tube formation of HUVECs in vitro

**Figure 3.**
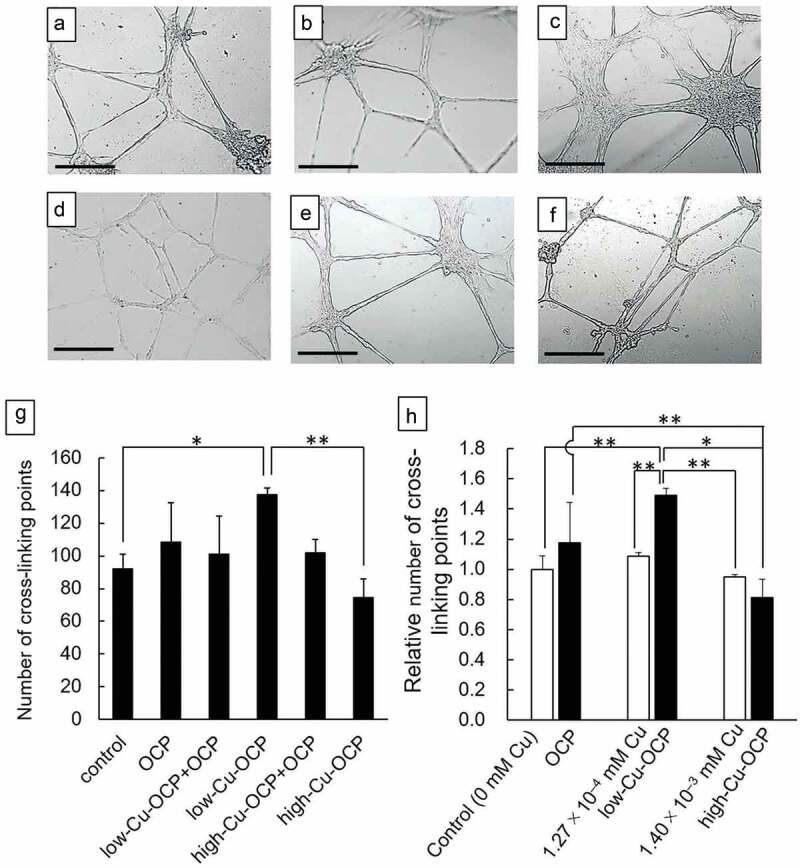
Optical microscope images of the capillary-like tubes formed by HUVECs incubated in the absence (control) (a) and the presence of 1 mg of OCP (b), low-Cu-OCP + OCP (c), low-Cu-OCP (d), high-Cu-OCP + OCP (e), and high-Cu-OCP (f) at 24 hours. Bars in the images represent 250 μm. Quantitative analysis of the number of cross-liking points in the tube networks of HUVECs cultured with 1 mg of granules containing different amounts of Cu (g). Relative number of cross-linking points in the networks of HUVECs cultured with OCP, low-Cu-OCP, high-Cu-OCP and copper gluconate at 0 (control), 1.27 × 10^–4^, and 1.40 × 10^–3^ mM (h). (*p < 0.05 and **p < 0.01).

The effects of the Cu content in granules on vascularization was examined by the formation of endothelial tubes in HUVECs in vitro (Figure 3). The cells formed a tube-like morphology in the absence (control group) and presence of granules regardless of the content of Cu after 24 hours of incubation. The capillary networks formed by HUVECs seemed to be better promoted in low-Cu-OCP (Figure 3(d)) groups compared to the control (Figure 3(a)), OCP (Figure3(b)), and low-Cu-OCP + OCP (Figure 3(c)). Network migration was observed to be more in high-Cu-OCP + OCP (Figure 3(e)) and high-Cu-OCP (Figure 3(f)) groups than in the low-Cu-OCP group (Figure 3(d)).

The formation of the tube networks was quantified by counting the cross-linking points in the networks (Figure 3(g)). The number of cross-linking points in the low-Cu-OCP group was significantly higher than those in the control and high-Cu-OCP groups. The number of cross-linking points tended to increase with the increasing Cu content in the OCP, low-Cu-OCP + OCP, and low-Cu-OCP groups. However, the number of points tended to decrease with the increasing Cu content in the low-Cu-OCP, high-Cu-OCP + OCP, and high-Cu-OCP groups. The cross-linking points in the high-Cu-OCP group were lower than those in the control group.

The formation of capillary network of HUVECs incubated in the presence of copper gluconate was also quantified (Figure 3(h)). The concentrations of copper gluconate was set at 1.27 × 10^–4^ and 1.40 × 10^–3^ mM, which corresponds to the Cu concentration in the media incubated with low-Cu-OCP and high-Cu-OCP granules, respectively (Table 3). The number of points in 1.27 × 10^–4^ mM group was slightly higher than that in control and 1.40 × 10^–3^ mM group, although the significant differences were not observed among these groups. The relative number of cross-linking points in low-Cu-OCP group was significantly higher than that in 1.27 × 10^–4^ mM as well as 1.40 × 10^–3^ mM groups. The relative number tended to decrease in high-Cu-OCP group compared to 1.40 × 10^–3^ mM group.

### 3.4. DNA concentration and ALP activity of D1 cell in vitro

**Figure 4.**
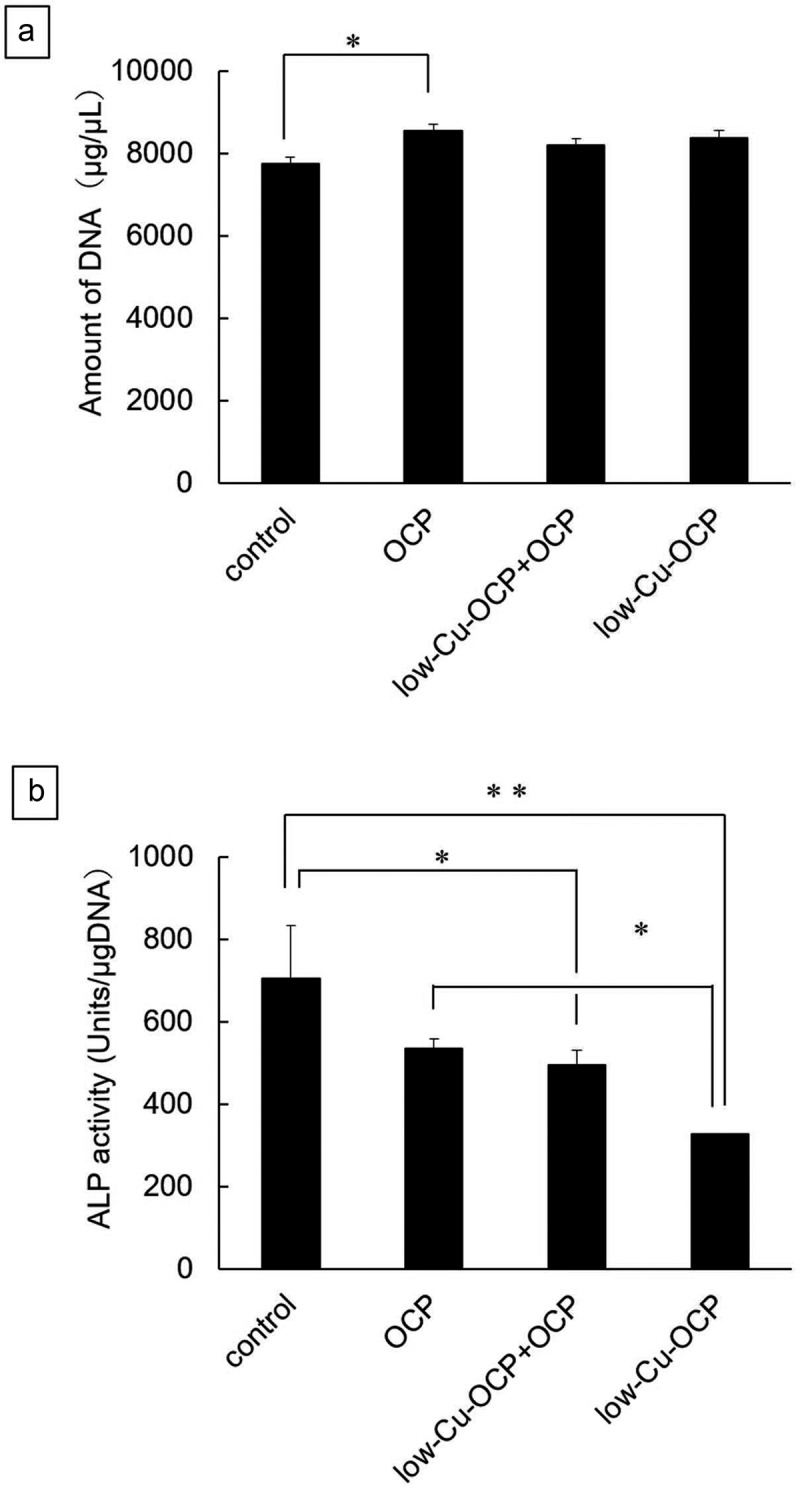
DNA amounts (a) and ALP activities (b) of D1 cells cultured in the absence (control) and the presence of 1 mg of OCP, low-Cu-OCP + OCP, and low-Cu-OCP at 14 days. (*p < 0.05 and **p < 0.01).

The behaviors of proliferation and osteoblastic differentiation in earlier stages of MSCs were examined by the culture of D1 cells in the absence (control group) or presence of the granules, including the lower content of Cu (Figure 4) at 14 days. The DNA concentration of cells indicating cell proliferation increased for all groups from 7 days to 14 days (Figure 4(a)). The DNA concentrations in the OCP, low-Cu-OCP + OCP, and low-Cu-OCP groups tended to be higher than those in the control group at 14 days. A significant difference was observed only between the control and OCP groups at 14 days. The ALP activity normalized by the DNA concentration in the control group was the highest among all groups at 14 days (Figure 4(b)). The activities tended to decrease with the increasing Cu content in the materials. Significant differences were observed between the control group and the low-Cu-OCP + OCP or low-Cu-OCP groups and between the OCP and low-Cu-OCP groups at 14 days.

### 3.5. Characterizations of OCP and Cu-OCP incubated with HUVECs and D1 cell in vitro

**Figure 5.**
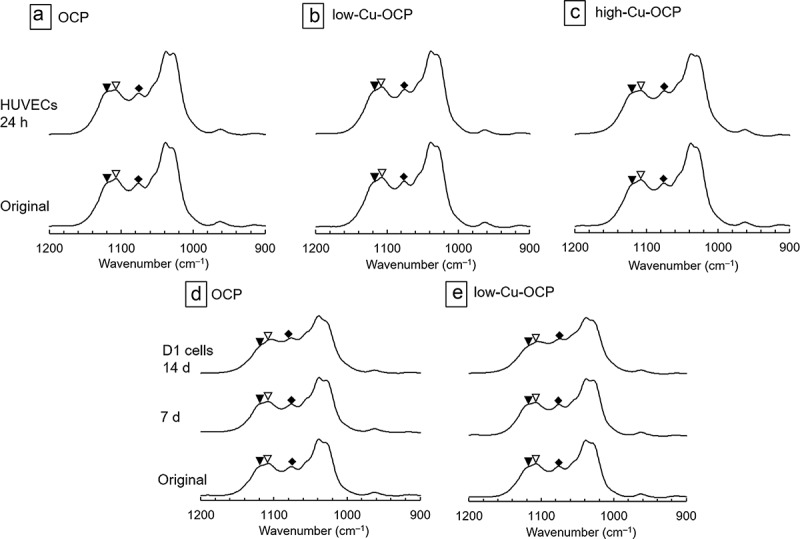
FTIR spectra of OCP (a, d), low-Cu-OCP (b, e), and high-Cu-OCP (c) before (original) and after incubations with HUVECs at 24 hours (a, b, c) and D1 cells at 14 days (d, e). Open and closed arrow heads indicate absorption bands of HPO_4_(5) and HPO_4_(6) vibrations at 1105 and 1121 cm^–1^, respectively. Rhombus indicates absorption bands of ν_3_ PO_4_ and ν_3_ HPO_4_ at 1070 cm^–1^.

The FTIR spectra of the granules before (original) and after incubation with HUVECs and D1 cells were measured to analyze the structural changes of OCP and Cu-OCP (Figure 5). The vibration bands corresponding to ν_3_ PO_4_ stretching were observed at 1025 and 1038 cm^–1^ in the FTIR spectra of the original OCP (Figure 5(a, d)), low-Cu-OCP (Figure 5(b,e)), and high-Cu-OCP (Figure 5(c)). The vibrations of ν_3_ PO_4_ and ν_3_ HPO_4_ clearly appeared in the spectra of all the original specimens at 1070 cm^–1^, which is a typical band of OCP. The ν (nu, Greek)_3_ HPO_4_(5) and ν (nu, Greek)_3_ HPO_4_(6) stretching vibrations were detected at 1105 and 1121 cm^–1^ in all original materials, which were attributed to HPO_4_^2–^ in the hydrated layer and at the junction of the apatite-like structure and the hydrated layer of OCP, respectively [41]. The intensities of ν (nu, Greek)_3_ HPO_4_(5) slightly decreased after incubation of all materials with HUVECs for 24 hours regardless of Cu content (Figure 5(a–c)). However, the intensity of ν (nu, Greek)_3_ HPO_4_(6) tended to maintain after the incubations of all specimens with HUVECs. The intensities of ν (nu, Greek)_3_ HPO_4_(6) also decreased after the incubations of OCP and low-Cu-OCP with D1 cells at 14 days. Furthermore, the intensities of ν (nu, Greek)_3_ HPO_4_(6) decreased compared to ν (nu, Greek)_3_ HPO_4_(6) in the spectra of all specimens incubated with D1 cell at 14 days. The absorption peak corresponding to ν_3_ PO_4_/ν_3_ HPO_4_ tended to be broad in the spectra of OCP and low-Cu-OCP after the incubations with D1 cells at 14 days.

**Figure 6.**
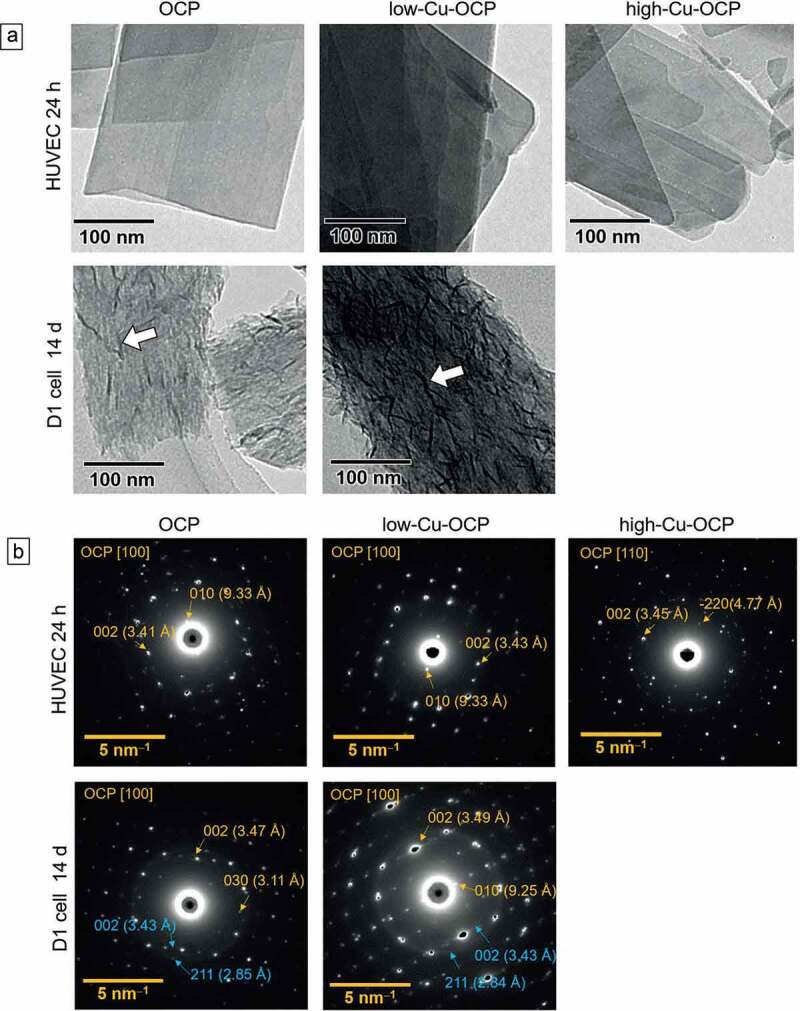
TEM images (a) and SAED patterns (b) of OCP, low-Cu-OCP, and high-Cu-OCP incubated with HUVECs at 24 hours and D1 cells at 14 days. Bars in the TEM images and SAED patterns represent 100 nm and 5 nm^–1^, respectively. Open allows in the TEM images indicate the de novo crystals formed on the OCP and low-Cu-OCP incubated with D1 cells at 14 days.

Morphological changes in OCP and Cu-OCP incubated with HUVECs and D1 cells were observed by TEM (Figure 6(a)). The plate-like morphologies of OCP, low-Cu-OCP, and high-Cu-OCP were maintained after incubation with HUVECs at 24 hours. On the surface of OCP and Cu-OCP, no deposition was observed after incubation with HUVECs. In contrast, depositions with a needle-like shape formed on the surface of OCP and low-Cu-OCP incubated with D1 cells at 14 days.

The crystal structures of OCP and Cu-OCP incubated with these cells were also characterized by SAED (Figure 6(b)). The diffraction spots of 010 and 002 were detected at 9.3 and 3.4 Å, respectively, in the patterns of OCP and low-Cu-OCP after being incubated with HUVECs at 24 hours. These patterns correspond to the zone axes of [100] of the OCP. In the pattern of high-Cu-OCP incubated with HUVECs, the spots attributed to $\overset{ˉ}{2}$20 and 002 were at 4.7 and 3.4 Å, respectively, which indicated the zone axis of [110] of OCP. In contrast, the diffraction rings corresponding to 002 and 211 of HA were newly detected at 3.4 and 2.8 Å, respectively, in the patterns of OCP and low-Cu-OCP after incubation with D1 cells at 14 days. The diffractions corresponding to the zone axes of [100] of the OCP were also observed in these patterns at 14 days. The intensity of ring diffraction in the pattern of low-Cu-OCP tended to be stronger than that in the pattern of OCP after 14 days of incubation, which indicated that a larger amount of HA was formed on low-Cu-OCP compared to OCP.

The results of the analyses and observations suggested that the hydrolysis reaction tended to progress with the increasing Cu content in OCP incubated with HUVECs and D1 cells.

### 3.6. Microstructure of OCP/Gel and low-Cu-OCP/Gel composites and Cu/Gel sponge prepared for implantation in vivo

**Figure 7.**
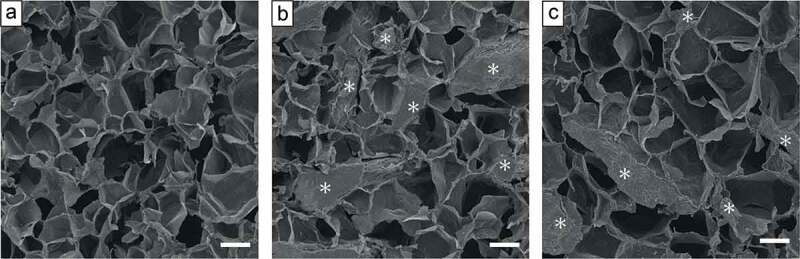
SEM images of the cross-sections of Cu/Gel (a), OCP/Gel (b), and low-Cu-OCP/Gel (c). Bars in the images represent 100 μm. Asterisks in the OCP/Gel and low-Cu-OCP/Gel indicate OCP and low-Cu-OCP granules, respectively.

Figure 7 shows SEM images of the cross-sections of OCP/Gel and low-Cu-OCP/Gel composites and Cu/Gel sponge. The Gel matrices of OCP/Gel and low Cu-OCP/Gel as well as Cu/Gel formed microporous structures. The average pore diameter was 165.8 ± 9.5, 161.9 ± 10.9, and 179.5 ± 6.5 μm, for Cu/Gel, OCP/Gel, and low-Cu-OCP/Gel, respectively (Table 4). The granules dispersed in the Gel matrixes were observed in the images of OCP/Gel and low-Cu-OCP/Gel. The depositions were not observed on the surface of Gel matrixes in Cu/Gel. These results suggest that OCP/Gel, low-Cu-OCP/Gel, and Cu/Gel possessed similar microstructures.

**Table 4.** Pore size of Cu/Gel, OCP/Gel, and low-Cu-OCP/Gel composite

Specimens	Average pore size (μm)
Cu/Gel	165.8 ± 9.5
OCP/Gel	161.9 ± 10.9
low-Cu-OCP/Gel	179.5 ± 6.5

### 3.7. Radiographic analysis of angiogenesis in the defect region treated with Cu/Gel, OCP/Gel, and low-Cu-OCP/Gel

**Figure 8.**
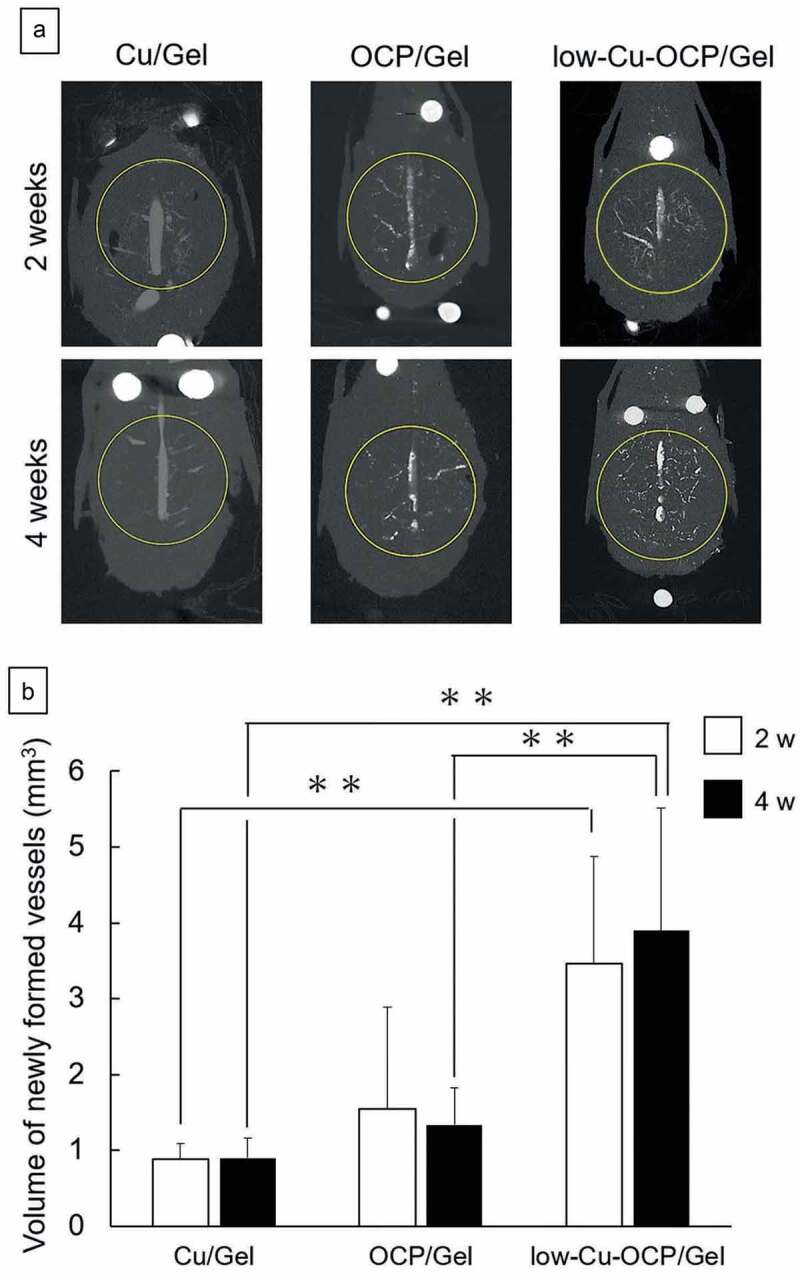
Micro-CT images of decalcified calvarial tissues around the region of defects treated with Cu/Gel, OCP/Gel, and low-Cu-OCP/Gel at 2 and 4 weeks of post-implantation (a). Inside of yellow circles in the micro-CT images indicates the region of defects. Quantitative analysis of volume of newly formed vessels in ROI of the calvarial defects treated with Cu/Gel, OCP/Gel, and low-Cu-OCP/Gel at 2 and 4 weeks of post-implantation. (*p < 0.05 and **p < 0.01).

The behaviors of angiogenesis and bone formation induced by the implantation of Cu/Gel sponge and OCP/Gel and low-Cu-OCP/Gel composites in the bone defects were examined by the micro-CT analysis of the calvarial tissues. The micro-CT images in Figure 8(a) and Figures S1, and S2 (in the supplementary information) show the cross-section in the transverse plane of the collected tissues after decalcification. The presence of an intact sagittal sinus visualized by the radiopaque agent was confirmed in the center of the defect region in all groups at 2 and 4 weeks. Thick newly formed vessels branching from the sagittal sinuses were also observed in all groups. At 2 weeks, tiny new vessels were distributed over the defect in the low-Cu-OCP/Gel group, while the formation of the tiny vessels seemed to be limited around the thick new vessels in the OCP/Gel groups. In contrast, there were only a few tiny vessels that could be clearly observed in the Cu/Gel group at 2 weeks. The distributions of the new tiny vessels at 4 weeks displayed a tendency similar to that at 2 weeks.

The volume of the newly formed vessels in the defect region was quantified by the 3D analysis of the micro-CT images of the collected tissues after decalcification (Figure 8(b)). At 2 and 4 weeks, the volumes of newly formed vessels in the low-Cu-OCP/Gel and OCP/Gel groups were larger than those in Cu/Gel groups, while the values increased in the low-Cu-OCP/Gel group compared to the OCP/Gel group. Significant differences were observed between the low-Cu-OCP/Gel group and OCP/Gel or Cu/Gel group at 4 weeks, and between Cu/Gel and low-Cu-OCP/Gel groups at 2 weeks. The volume of newly formed vessels slightly increased in the low-Cu-OCP/Gel group from 2 to 4 weeks. However, the volume in the OCP/Gel group at 4 weeks was lower than that at 2 weeks. The lower volume in the Cu/Gel group was maintained from 2 to 4 weeks.

### 3.8. Radiographic analysis of new calcified tissues in the defect region treated with Cu/Gel, OCP/Gel, and low-Cu-OCP/Gel

**Figure 9.**
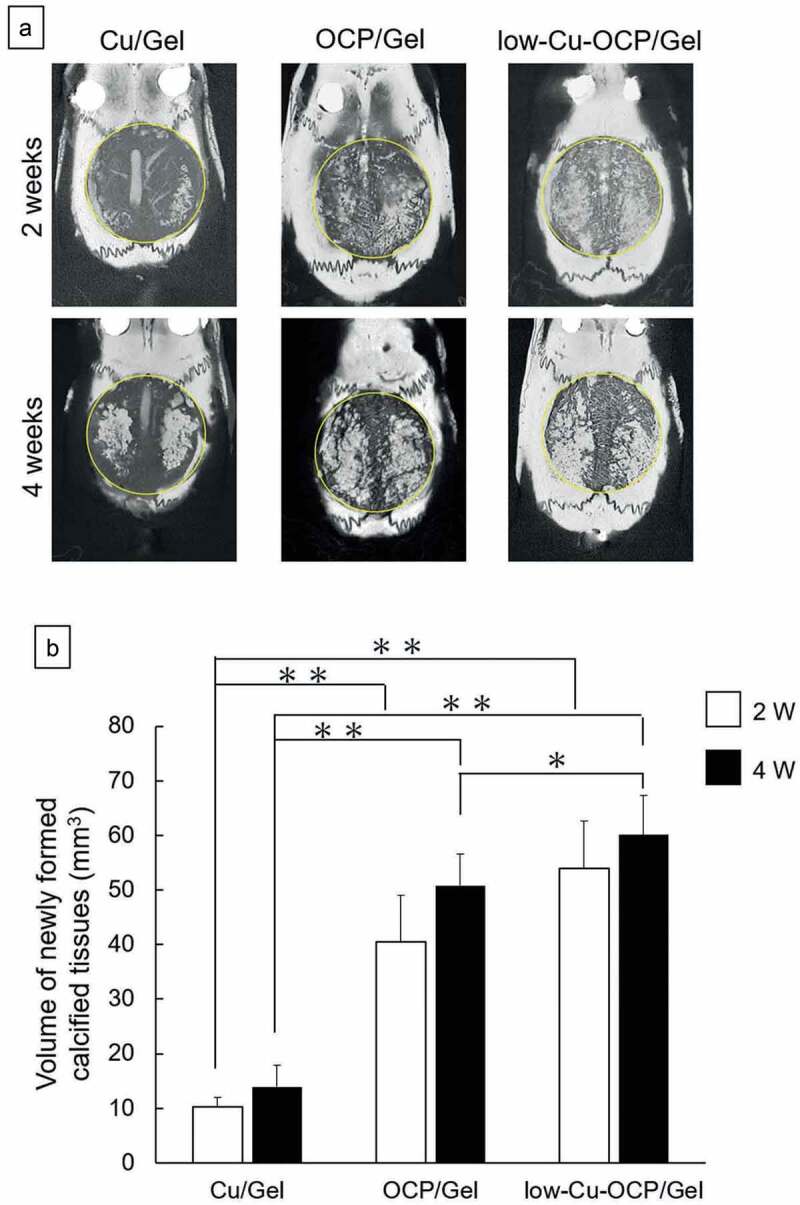
Micro-CT images of undecalcified calvarial tissues around the region of defects treated with Cu/Gel, OCP/Gel, and low-Cu-OCP/Gel at 2 and 4 weeks of post-implantation (a). Inside of yellow circles in the micro-CT images indicates the region of defects. Quantitative analysis of volume of newly formed calcified tissues in ROI of the calvarial defect treated with Cu/Gel, OCP/Gel, and low-Cu-OCP/Gel at 2 and 4 weeks of post-implantation. (*p < 0.05 and **p < 0.01).

The bone formations in the defect regions treated with Cu/Gel, OCP/Gel, and low-Cu-OCP/Gel were visualized in the micro-CT images of the cross-sections of the collected tissues before decalcification (Figure 9(a) and Figures S3 and S4 in the Supplementary Information). Radiopaque regions indicating newly formed calcified tissues were observed in the defects in the OCP/Gel and low-Cu-OCP/Gel groups at 2 weeks. The area of the radiopaque region connecting with the edge of the defect seemed to be larger in the low-Cu-OCP group than in the OCP/Gel group at 2 weeks. In contrast, the region of radiopacity was limited around the edge of the defect in the Cu/Gel group at 2 weeks. The radiopacity levels and radiopacity regions in the OCP/Gel and low-Cu-OCP groups tended to increase at 4 weeks compared to those at 2 weeks. Moreover, a radiopacity region was observed around the edge of the defect in the Cu/Gel group at 4 weeks.

The volume of the newly formed calcified tissues in the defect region was calculated by the 3D analysis of the micro-CT images (Figure 9(b)). The volumes of the newly formed calcified tissue tended to increase over 2 weeks to 4 weeks after implantation regardless of the materials, although there were no significant differences. At 2 and 4 weeks, the volumes of the calcified tissue in the low-Cu-OCP and OCP/Gel groups were significantly larger than those in the Cu/Gel group. Although the volume of the calcified tissue in low-Cu-OCP also increased compared to that in the OCP/Gel group at 2 and 4 weeks, a significant difference was only observed at 4 weeks.

### 3.9. Histological and histomorphometric analyses of newly formed tissue in the defect region treated with Cu/Gel, OCP/Gel, and low-Cu-OCP/Gel

**Figure 10.**
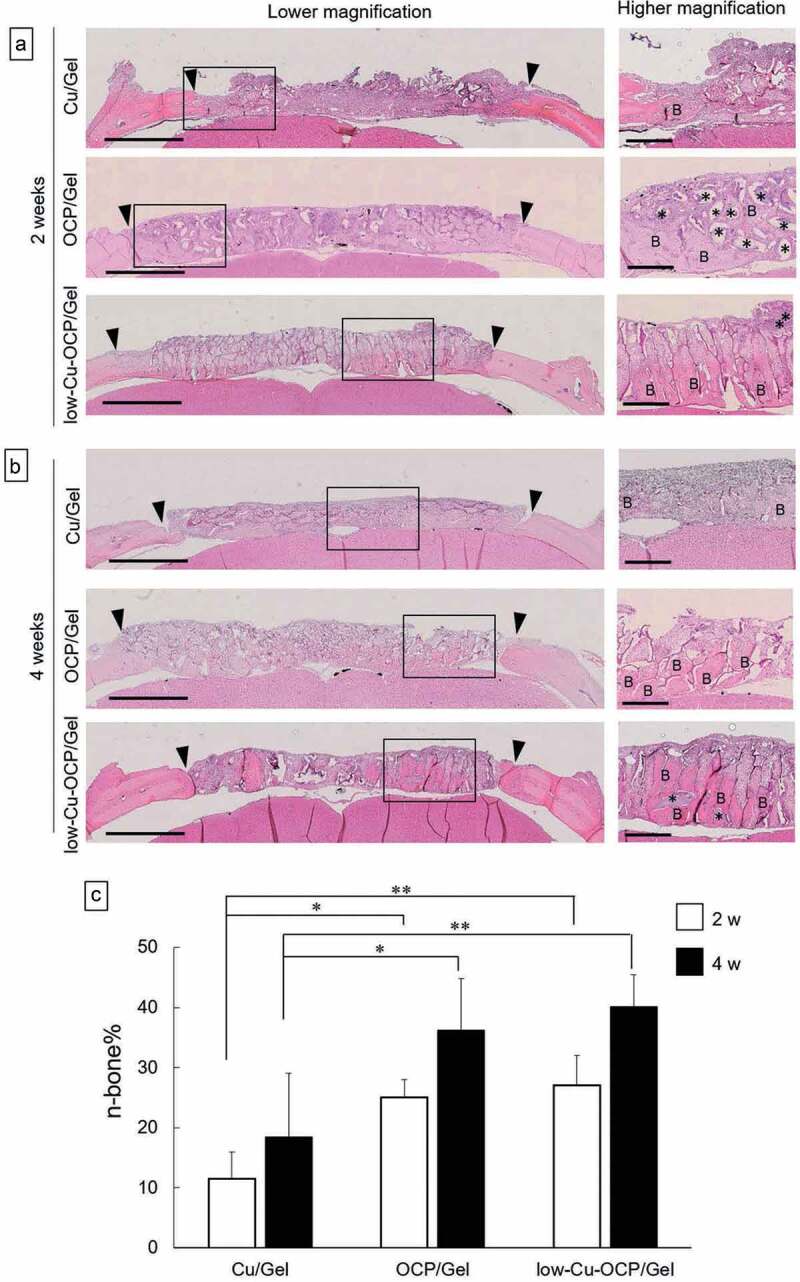
Histological sections with the H&E staining of rat calvaria defects treated with the implantation of Cu/Gel, OCP/Gel, and low-Cu-OCP/Gel at 2 (a) and 4 weeks (b). Open squares indicate the areas shown as high magnification images. Bars in the low and high magnification images represent 2 mm and 500 μm, respectively. Arrow heads indicate the edges of bone defects. Asterisks and ‘B’ indicate remaining granules of OCP or low-Cu-OCP and newly formed bone, respectively. Histomorphometric analysis of the newly formed bone area in the bone defect region treated with Cu/Gel, OCP/Gel, and low-Cu-OCP/Gel at 2 and 4 weeks post-implantation (c). (*p < 0.05, **p < 0.01).

The behavior of new bone tissue formation in the defects was also examined by histological analysis using the H&E staining of the sections (Figure 10(a,b)). The low magnification images show that continuous new bone tissues stained by H&E were formed from the edge of the defect in the OCP/Gel and low-Cu-OCP/Gel at 2 weeks (Figure 10(a)). The remaining granules stained with hematoxylin were detected in the defect regions. The remaining granules that were in direct contact with the new bone tissues were observed in OCP/Gel and Cu-OCP/Gel groups at 2 weeks in the high magnification images. On the contrary, the connective tissues were distributed over the defect treated with Cu/Gel for 2 weeks.

The regions of newly formed tissues growing toward the center of the defect were observed in OCP/Gel and low-Cu-OCP/Gel at 4 weeks in the low magnification images, although the connective tissue seemed to remain at the center of the defect (Figure 10(b)). The high magnification images show that the thickness of new bone tissues tended to increase in OCP/Gel and low-Cu-OCP/Gel at 4 weeks compared to those at 2 weeks (Figure 10(b)). Thin new bone tissues formed from the edge of the defect in the Cu/Gel group at 4 weeks (Figure 10(b)).

The formation ratio of the new bone tissue (n-bone%) in the defect area was quantified by histomorphometric analysis at 2 and 4 weeks (Figure 10(c)). The values of n-bone% at 4 weeks tended to be higher than those at 2 weeks in all groups, although a significant difference was not observed between 2 and 4 weeks in each group. The n-bone% values in OCP/Gel and low-Cu-OCP/Gel groups significantly increased compared to those of Cu/Gel group at 2 and 4 weeks, while the n-bone% in low-Cu-OCP/Gel group was slightly higher than that in OCP/Gel group at each implantation period. However, there were no significant differences between the OCP/Gel and low-Cu-OCP/Gel groups at 2 and 4 weeks.

### 3.10. Immunostaining of endomucin and VEGF

**Figure 11.**
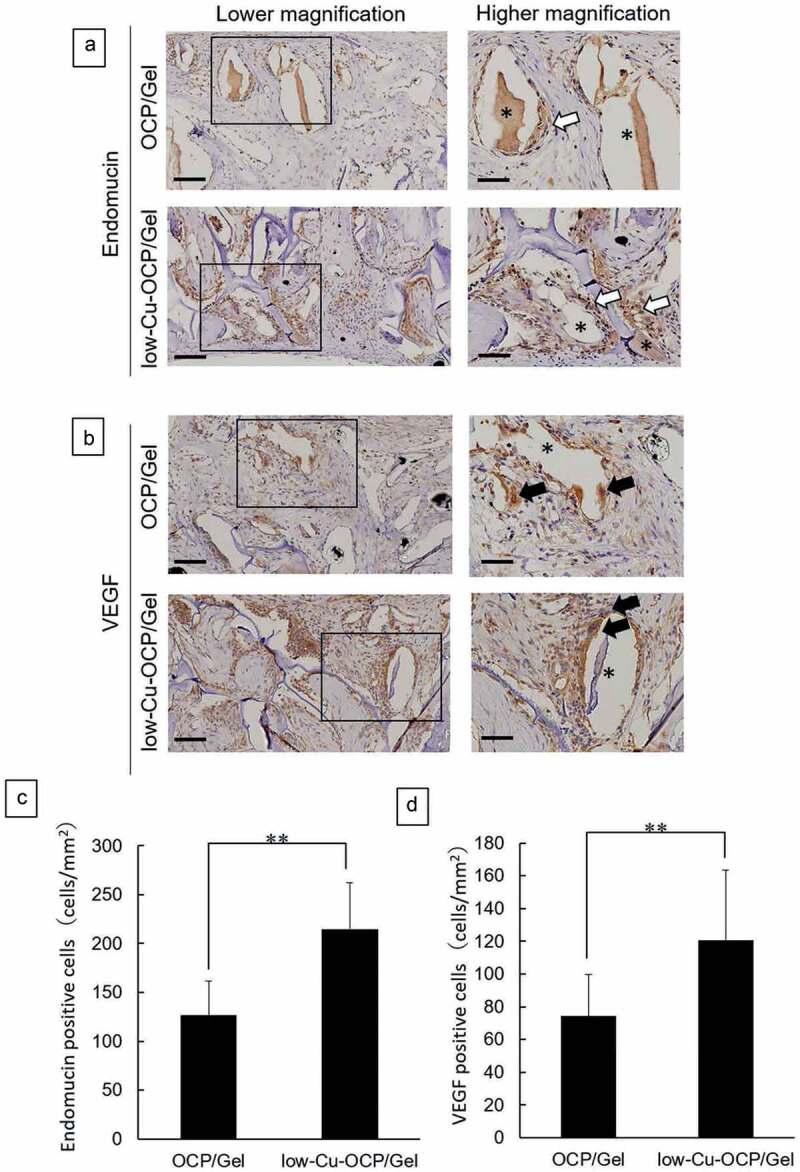
Immunostaining of endomucin (a) and VEGF (b) of the sections at 4 weeks after the implantation of OCP/Gel and low-Cu-OCP/Gel. Open squares indicate the areas shown as high magnification images. Bars in the low and high magnification images represent 100 and 50 μm, respectively. Open and closed arrows indicate endomucin- and VEGF- positive cells. Asterisks indicate remaining granules of OCP or low-Cu-OCP. Quantitative analysis of endomucin- (c) and VEGF- (d) positive cells in the unit area of ROI. (**p < 0.01).

The presence of newly formed vessels was examined by immunostaining for endomucin (Figure 11(a)). In addition, the cells that expressed the factors involved in the promotion of angiogenesis and osteogenesis were detected by immunostaining for VEGF (Figure 11(b)). Endomucin-positive cells were observed around the remaining granules in the defects treated with OCP/Gel and low-Cu-OCP/Gel composites at 4 weeks (Figure 11(a)). The VEGF-positive cells also appeared to be present around the granules in the defects after the implantation of OCP/Gel and low-Cu-OCP/Gel at 4 weeks (Figure 11(b)). The number of endomucin- (Figure 11(c)) and VEGF- (Figure 11(d)) positive cells per unit area in the defect was determined at 4 weeks. The number of endomucin-positive cells in the low-Cu-OCP/Gel group was significantly higher than that in the OCP/Gel group. Moreover, the number of VEGF-positive cells significantly increased in the low-Cu-OCP/Gel group compared to that in the OCP/Gel group.

## 4. Discussion

The present study revealed that the OCP/Gel with a low copper content promoted angiogenesis and osteogenesis earlier than the copper-free OCP/Gel. The culture of HUVECs showed that low-Cu-OCP advanced the capillary-like tube formation of HUVECs more than OCP (Figure 3). In the culture of D1 cells, low-Cu-OCP showed lower ALP activity than OCP (Figure 4(b)), suggesting that Cu^2+^ may have inhibited initial osteoblastic differentiation. From the DS in the cell culture media and XRD analysis, Cu-OCP became more soluble than OCP in a dose-dependent manner (Tables 1 and 3). The FTIR and TEM results before and after immersion in the media showed that the hydrolysis of Cu-OCP was more accelerated than that of OCP (Figures 5 and 6). In rat calvarial bone defects, low-Cu-OCP/Gel promoted better neovascularization compared to OCP/Gel, activated VEGF expression, and resulted in more bone formation (Figures 8–11).

Cu^2+^ has been proven to be beneficial for blood vessels, and the concentration of Cu^2+^ influences the response of HUVECs [42,43]. Gerald et al. reported that the formation of globular and/or tubular structures of HUVECs was promoted with CuSO_4_ at 50 μg/mL (= 50 mg/L) [42]. Shahli et al. cultured HUVECs in two types of medium: normal medium and nutrient-reduced medium [42]. They revealed that in normal medium, the metabolic activity and viability of HUVECs decreased in a dose-dependent manner at 0.44 to 12 ppm (= 0.44 to 12 mg/L). In contrast, in the nutrient-reduced medium, the highest metabolic activity of HUVECs was observed at 4 ppm (4 mg/L). In this study, the Cu^2+^ concentrations released by low-Cu-OCP and high-Cu-OCP were 1.27 × 10^–4^ mM (= 0.008 mg/L) and 1.40 × 10^–3^ mM (= 0.089 mg/L), respectively, which were much lower than those reported in previous studies (Table 3). The lumen formation of HUVECs was most enhanced in the presence of low-Cu-OCP, when the concentration of Cu^2+^ was 0.004 mg/L (Figure 3(g)). This suggests that the optimal Cu content in OCPs may promote lumen formation in HUVECs.

The Cu^2+^ released from low-Cu-OCP significantly enhanced the formation of capillary networks of HUVECs compared to Cu^2+^ derived from copper gluconate, although these concentrations were comparable level (Figure 3(h)). In the culture media, Ca^2+^ and Pi ion concentrations also increased after the incubations with low-Cu-OCP and high-Cu-OCP (Table 3). The previous literatures indicated that the dissolved ions from calcium phosphates such as β-TCP [44] and OCP [18] enhanced the vascularization in the absence of foreign ions in these calcium phosphates in vitro. However, Cu^2+^ dissolved from high-Cu-OCP, which exhibited the highest solubility (Table 3), tended to suppress the capillary formation compared to copper gluconate-derived Cu^2+^ at the same concentration (Figure 3(h)). Taken together, the ionic environment including the lower Cu^2+^ concentration regulated by the dissolution of low-Cu-OCP could be suitable to promote the capillary formation in vitro.

Cu^2+^ concentration affects the viability of MSCs [45]. The viability of rat bone marrow MSCs decreases at CuSO_4_ concentrations above 10 μM (= 0.636 mg/L) [45]. In this study, there was no significant difference in the DNA concentration of mouse bone marrow MSCs (D1) between the OCP and low-Cu-OCP groups, suggesting that Cu^2+^ concentrations as low as 0.004 mg/L did not affect mouse MSC survival (Figure 4(a)).

The effects of Cu^2+^ ions on osteoblastic differentiation remains controversial. Rodriguez et al. reported that Cu^2+^ at 50 μM (= 3.18 mg/L) inhibited the proliferation and promoted the osteoblastic differentiation of human MSCs [46]. Furthermore, Li et al. reported that 5 μM Cu ion (= 0.32 mg/L) inhibited the ALP activity, expression of osteogenic genes, and formation of bone nodules in rat MSCs [45]. In the present study, the ALP activity of mouse MSCs (D1) was suppressed with low-Cu-OCP (Figure 4(b)) when the Cu^2+^ concentration was 0.008 mg/L and even lower than that in previous studies (Table 3).

We have previously shown that OCP crystals containing various ions, such as F ^–^ and Zn^2+^, affect their solubility and hydrolysis from OCP to HA [9,47]. Differences in the solubility of OCP have also been confirmed to affect the bone regeneration ability in mouse calvaria and rat tibia [33]. Therefore, we analyzed the solubility of Cu-OCP by the DS in the culture medium and found that Cu-OCP showed a higher solubility than OCP in a dose-dependent manner. Previous studies revealed that synthesis in the presence of metal ions affects three factors: 1) the lattice contraction and enlargement of the calcium phosphates, 2) the decrease in particle size, and 3) crystallinity [48]. These factors modulate the solubility of calcium phosphate crystals [48,49]. Rietveld analysis showed that the crystal lattice of OCP decreased with the increasing Cu content in the OCP (Table 1). The ionic radius of Cu^2+^ (0.073 nm) is smaller than that of Ca^2+^ (0.099 nm), suggesting that the substitution of Ca^2+^sites with Cu^2+^ in the lattice may have caused the crystal lattice of Cu-OCP to contract. The Cu could be present not only in the crystal, but also adsorbed on the surface of crystal. However, the lattice contract seemed to be greater versus the content of Cu in the specimens in this study. Our previous report suggests that the lattice of OCP tended to be contracted by the dislocation incorporated during the crystal growth in the presence of organic molecules [50]. The decrease in lattice parameters for Cu-OCP prepared using copper gluconate could be also associated with the incorporation of lattice defects except the substitution of foreign ion, but the detail mechanism is still unclear why the greater lattice contract in the range of lower content of Cu in OCP took place. We previously reported that the synthesis of OCP in the presence of Zn^2+^ resulted in a decrease in the particle size when the Zn^2+^ concentration was above 1.3 mM [9]. However, Cu-OCP synthesized in the presence of low concentrations of copper gluconate (0.0025–0.025 mM) did not exhibit a significant suppression of crystal growth (Figure 1). In addition, the values of FWHM suggest that crystallinity of OCP could not be regulated dominantly by the content of Cu up to 0.12 wt.% (Figure 1). From these results, lattice contraction is expected to be one of the main reasons why Cu-OCP showed a higher solubility than OCP. The elongation of the crystal lattice increases the solubility of HA and α-tricalcium phosphate (α-TCP) by distorting the lattice [49,51]. The lattice distortion caused by the incorporation of line defects increases the solubility of OCP and accelerates its hydrolysis to HA [50]. In other words, the lattice distortion that led to lattice contraction may be related to the high solubility of Cu-OCP. The higher solubility of Cu-OCP resulted in a more saturated environment for HA (Table 3), suggesting that hydrolysis is more likely to occur in Cu-OCP than in OCP.

Cu^2+^ improves angiogenesis and osteogenesis in vivo. Gérard et al. reported that the subcutaneous implantation of cylindrical collagen scaffolds with CuSO_4_ increased the number of CD31-positive microvessels and promoted collagen deposition [42]. We have previously shown that the OCP/Gel composite promotes angiogenesis and osteogenesis in rat calvarial defects compared to Gel alone [18]. To clarify the angiogenic and osteogenic potential of low-Cu-OCP, we implanted three types of materials, low-Cu-OCP/Gel, OCP/Gel, and Gel containing the same Cu^2+^ concentration as low-Cu-OCP (Cu/Gel), into the rat calvarial defect. At 4 weeks after implantation, the volume of neovascularization was significantly higher in the low-Cu-OCP/Gel than those in the Cu/Gel and OCP/Gel (Figure 8(b)). Significantly, Cu/Gel did not promote angiogenesis, suggesting that combining Cu^2+^ with OCP is an important pathway for inducing vessel formation. In immunostaining, more endomucin-positive cells were observed around low-Cu-OCP/Gel than OCP/Gel, confirming the promotion of angiogenesis by low-Cu-OCP in vivo and in vitro.

Although low-Cu-OCP inhibited the differentiation of mouse MSCs in vitro (Figure 4(b)), low-Cu-OCP/Gel showed the highest bone formation in rat calvarial defects (Figures 9(b) and 10(b)). Therefore, considering the possibility of indirect bone formation by other bone-related cells, except osteoblasts, we performed immunostaining for VEGF. The results showed that low-Cu-OCP/Gel significantly increased the number of VEGF-positive cells compared to OCP/Gel after 4 weeks of implantation (Figure 11(b,d)). Studies by other groups have also reported that Cu^2+^ increases the expression of VEGF, which is an important factor in the coupling of angiogenesis and osteogenesis [52,53]. VEGF promotes osteoblast migration [54,55] and differentiation [56]. As VEGF has also been reported to enhance osteoclast survival and resorption [57], low-Cu-OCP/Gel may have activated bone remodeling by osteoblasts and osteoclasts via VEGF leading to enhanced bone regeneration in rat calvarial defects.

## 5. Conclusions

The results of this study indicate that a complex capillary-like tube network of HUVECs was formed in the presence of low-Cu-OCP (0.01 wt.% Cu) prepared by the co-precipitation with copper gluconate compared to OCP in vitro, although the osteoblastic differentiation of MSCs was inhibited by low-Cu-OCP. However, low-Cu-OCP/Gel composite exhibited the higher capacities of angiogenesis and osteogenesis than OCP/Gel composite in the rat calvarial critical sized bone defect while it increased the expression of VEGF. In physiological environment, the higher solubility of low-Cu-OCP accelerated the hydrolysis reaction to transform into HA. The present study suggests that the bone regeneration via remarkable angiogenesis could be induced thanks to the suitable Cu^2+^ release and physiochemical stability of low-Cu-OCP.

## Supplementary Material

Supplemental Material

## References

[1] Brown E, Walter JPS, Lehr JR, et al. Octacalcium phosphate and hydroxyapatite: crystallographic and chemical relations between octacalcium phosphate and hydroxyapatite. Nature. 1962;196:1050–1055.

[2] Brown WE. Crystal growth of bone mineral. Clin Orthop Relat Res. 1966;44:205–220. 5910250

[3] Simon P, Grüner D, Worch H, et al. First evidence of octacalcium phosphate@osteocalcin nanocomplex as skeletal bone component directing collagen triple-helix nanofibril mineralization. Sci Rep. 2018;8(1):13696. DOI:10.1038/s41598-018-31983-5 30209287 PMC6135843

[4] Mathew M, Brown WE, Schroeder LW, et al. Crystal structure of octacalcium bis(hydrogenphosphate) tetrakis(phosphate) pentahydrate, Ca_8_(HPO_4_)_2_(PO_4_)_4_·5H_2_O. J Crystallogr Spectrosc Res. 1988;18:235–250.

[5] Suzuki O, Yagishita H, Amano T, et al. Reversible structural changes of octacalcium phosphate and labile acid phosphate. J Dent Res. 1995;74(11):1764–1769. DOI:10.1177/00220345950740110801 8530738

[6] Miyatake N, Kishimoto KN, Anada T, et al. Effect of partial hydrolysis of octacalcium phosphate on its osteoconductive characteristics. Biomaterials. 2009;30(6):1005–1014. DOI:10.1016/j.biomaterials.2008.10.058 19027945

[7] Habraken WJ, Tao J, Brylka LJ, et al. Ion-association complexes unite classical and non-classical theories for the biomimetic nucleation of calcium phosphate. Nat Commun. 2013;4:1507. 23422675 10.1038/ncomms2490

[8] Matsunaga K. First-principles study of substitutional magnesium and zinc in hydroxyapatite and octacalcium phosphate. J Chem Phys. 2008;128(24):245101. 18601385 10.1063/1.2940337

[9] Honda Y, Anada T, Morimoto S, et al. Effect of Zn^2+^ on the physicochemical characteristics of octacalcium phosphate and its hydrolysis into apatitic phases. Cryst Growth Des. 2011;11(5):1462–1468. DOI:10.1021/cg1009835

[10] Shi H, He F, Ye J. Synthesis and structure of iron- and strontium-substituted octacalcium phosphate: effects of ionic charge and radius. J Mater Chem B. 2016;4(9):1712–1719. 32263022 10.1039/c5tb02247a

[11] Sugiura Y, Obika H, Horie M, et al. Aesthetic silver-doped octacalcium phosphate powders exhibiting both contact antibacterial ability and low cytotoxicity. ACS Omega. 2020;5(38):24434–24444. DOI:10.1021/acsomega.0c02868 33015459 PMC7528307

[12] Suzuki O, Shiwaku Y, Hamai R. Octacalcium phosphate bone substitute materials: comparison between properties of biomaterials and other calcium phosphate materials. Dent Mater J. 2020;39(2):187–199. 32161239 10.4012/dmj.2020-001

[13] Suzuki O, Nakamura M, Miyasaka Y, et al. Bone formation on synthetic precursors of hydroxyapatite. Tohoku J Exp Med. 1991;164(1):37–50. DOI:10.1620/tjem.164.37 1926145

[14] Suzuki O, Kamakura S, Katagiri T, et al. Bone formation enhanced by implanted octacalcium phosphate involving conversion into Ca-deficient hydroxyapatite. Biomaterials. 2006;27(13):2671–2681. DOI:10.1016/j.biomaterials.2005.12.004 16413054

[15] Komlev VS, Barinov SM, Bozo II, et al. Bioceramics composed of octacalcium phosphate demonstrate enhanced biological behavior. ACS Appl Mater Interfaces. 2014;6(19):16610–16620. DOI:10.1021/am502583p 25184694

[16] Sugiura Y, Munar ML, Ishikawa K. Fabrication of octacalcium phosphate block through a dissolution-precipitation reaction using a calcium sulphate hemihydrate block as a precursor. J Mater Sci Mater Med. 2018;29(10):151. 30264167 10.1007/s10856-018-6162-1

[17] Kim J, Kim S, Song I. Biomimetic octacalcium phosphate bone has superior bone regeneration ability compared to xenogeneic or synthetic bone. Materials. 2021;14(18):5300. 10.3390/ma14185300 PMC8470492 34576527

[18] Kurobane T, Shiwaku Y, Anada T, et al. Angiogenesis involvement by octacalcium phosphate-gelatin composite-driven bone regeneration in rat calvaria critical-sized defect. Acta Biomater. 2019;88:514–526. 30776505 10.1016/j.actbio.2019.02.021

[19] Hench LL, Xynos ID, Polak JM. Bioactive glasses for in situ tissue regeneration. J Biomater Sci Polym Ed. 2004;15(4):543–562. 15212333 10.1163/156856204323005352

[20] Kawamura H, Ito A, Miyakawa S, et al. Stimulatory effect of zinc-releasing calcium phosphate implant on bone formation in rabbit femora. J Biomed Mater Res. 2000;50(2):184–190. DOI:10.1002/(SICI)1097-4636(200005)50:2<184::AID-JBM13>3.0.CO;2-3 10679683

[21] Takami M, Mochizuki A, Yamada A, et al. Osteoclast differentiation induced by synthetic octacalcium phosphate through receptor activator of NF-κB ligand expression in osteoblasts. Tissue Eng Part A. 2009;15(12):3991–4000. DOI:10.1089/ten.tea.2009.0065 19594360

[22] Sai Y, Shiwaku Y, Anada T, et al. Capacity of octacalcium phosphate to promote osteoblastic differentiation toward osteocytes in vitro. Acta Biomater. 2018;69:362–371. 29378325 10.1016/j.actbio.2018.01.026

[23] Saghiri MA, Asatourian A, Orangi J, et al. Functional role of inorganic trace elements in angiogenesis-Part II: cr, Si, Zn, Cu, and S. Crit Rev Oncol Hematol. 2015;96(1):143–155. 26088455 10.1016/j.critrevonc.2015.05.011

[24] Barralet J, Gbureck U, Habibovic P, et al. Angiogenesis in calcium phosphate scaffolds by inorganic copper ion release. Tissue Eng Part A. 2009;15(7):1601–1609. DOI:10.1089/ten.tea.2007.0370 19182977

[25] Monma H, Ueno S. The uptake of cadmium (II) and copper (II) ions by calcium phosphates. Nishikaishi. 1982;11:1859–1862.

[26] Chen J, Chen C, Wu Y, et al. Synthesis and characterization of copper ions doped octacalcium phosphate powders with enhanced osteogenic property. J Biomater. 2021;5(1):10–15. DOI:10.11648/j.jb.20210501.12

[27] Moradian-Oldak J, Iijima M, Bouropoulos N, et al. Assembly of amelogenin proteolytic products and control of octacalcium phosphate crystal morphology. Connect Tissue Res. 2003;44(1):58–64. DOI:10.1080/03008200390152106 12952175

[28] Bigi A, Boanini E, Walsh D, et al. Morphosynthesis of octacalcium phosphate hollow microspheres by polyelectrolyte-mediated crystallization. Angew Chem Int Ed Engl. 2002;41(12):2163–2166. DOI:10.1002/1521-3773(20020617)41:12<2163::AID-ANIE2163>3.0.CO;2-G 19746631

[29] Iijima M, Moriwaki Y, Yamaguchi R, et al. Effect of solution pH on the calcium phosphates formation and ionic diffusion on and through the collagenous matrix. Connect Tissue Res. 1997;36(2):73–83. DOI:10.3109/03008209709160215 9298625

[30] Handa T, Anada T, Honda Y, et al. The effect of an octacalcium phosphate co-precipitated gelatin composite on the repair of critical-sized rat calvarial defects. Acta Biomater. 2012;8(3):1190–1200. 22198138 10.1016/j.actbio.2011.12.002

[31] Shelton RM, Liu Y, Cooper PR, et al. Bone marrow cell gene expression and tissue construct assembly using octacalcium phosphate microscaffolds. Biomaterials. 2006;27(14):2874–2881. DOI:10.1016/j.biomaterials.2005.12.031 16439012

[32] Liu Y, Cooper PR, Barralet JE, et al. Influence of calcium phosphate crystal assemblies on the proliferation and osteogenic gene expression of rat bone marrow stromal cells. Biomaterials. 2007;28(7):1393–1403. DOI:10.1016/j.biomaterials.2006.11.019 17166582

[33] Shiwaku Y, Hamai R, Sato S, et al. Bone tissue response to different grown crystal batches of octacalcium phosphate in rat long bone intramedullary canal area. Int J Mol Sci. 2021;22(18):9770. DOI:10.3390/ijms22189770 34575928 PMC8466561

[34] Black RD, Windover D, Henins A, et al. Certification of NIST standard reference material 640d. Powder Diffr. 2010;25(2):187–190. DOI:10.1154/1.3409482

[35] Dasgupta S, Banerjee SS, Bandyopadhyay A, et al. Zn- and Mg-doped hydroxyapatite nanoparticles for controlled release of protein. Langmuir. 2010;26(7):4958–4964. DOI:10.1021/la903617e 20131882 PMC2862579

[36] Moreno EC, Kresak M, Zahradnik RT. Fluoridated hydroxyapatite solubility and caries formation. Nature. 1974;247(5435):64–65. 4462607 10.1038/247064a0

[37] Tung MS, Eidelman N, Sieck B, et al. Octacalcium phosphate solubility product from 4 to 37 °C. J Res Natl Bur Stand. 1988;93(5):613–624.

[38] Edgard C, Moreno WEB, Osborn G. Solubility of dicalcium phosphate dihydrate in aqueous systems. Soil Sci Soc Am J. 1960;24(2):94–98.

[39] Saito K, Anada T, Shiwaku Y, et al. Dose-dependent enhancement of octacalcium phosphate biodegradation with a gelatin matrix during bone regeneration in a rabbit tibial defect model. RSC Adv. 2016;6(69):64165–64174. DOI:10.1039/C6RA07602E

[40] Itagaki T, Honma T, Takahashi I, et al. Quantitative analysis and localization of mRNA transcripts of type I collagen, osteocalcin, MMP 2, MMP 8, and MMP 13 during bone healing in a rat calvarial experimental defect model. Anat Rec (Hoboken). 2008;291(8):1038–1046. DOI:10.1002/ar.20717 18615687

[41] Fowler BO, Markovic M, Brown WE. Octacalcium phosphate. 3. Infrared and Raman vibrational spectra. Chem Mater. 1993;5:1417–1423.

[42] Gérard C, Bordeleau LJ, Barralet J, et al. The stimulation of angiogenesis and collagen deposition by copper. Biomaterials. 2010;31(5):824–831. 19854506 10.1016/j.biomaterials.2009.10.009

[43] Stähli C, Muja N, Nazhat SN. Controlled copper ion release from phosphate-based glasses improves human umbilical vein endothelial cell survival in a reduced nutrient environment. Tissue Eng Part A. 2013;19(3–4):548–557. 23298240 10.1089/ten.tea.2012.0223

[44] Chen Y, Wnag J, Zhu XD, et al. Enhanced effect of β-tricalcium phosphate phase on neovascularization of porous calcium phosphate ceramics: in vitro and in vivo evidence. Acta Biomater. 2015;11:435–448. 25246313 10.1016/j.actbio.2014.09.028

[45] Li S, Wang M, Chen X, et al. Inhibition of osteogenic differentiation of mesenchymal stem cells by copper supplementation. Cell Prolif. 2014;47(1):81–90. DOI:10.1111/cpr.12083 24450813 PMC6496232

[46] Rodríguez JP, Ríos S, González M. Modulation of the proliferation and differentiation of human mesenchymal stem cells by copper. J Cell Biochem. 2002;85(1):92–100. 11891853

[47] Suzuki O, Yagishita H, Yamazaki M, et al. Adsorption of bovine serum albumin onto octacalcium phosphate and its hydrolyzates. Cells Mater. 1995;5(1):45–54.

[48] Boanini E, Gazzano M, Bigi A. Ionic substitutions in calcium phosphates synthesized at low temperature. Acta Biomater. 2010;6(6):1882–1894. 20040384 10.1016/j.actbio.2009.12.041

[49] Schumacher M, Gelinsky M. Strontium modified calcium phosphate cements - approaches towards targeted stimulation of bone turnover. J Mater Chem B. 2015;3(23):4626–4640. 32262477 10.1039/c5tb00654f

[50] Hamai R, Sakai S, Shiwaku Y, et al. Octacalcium phosphate crystals including a higher density dislocation improve its materials osteogenecity. Appl Mater Today. 2022;26:10279.

[51] Pina S, Torres PM, Goetz-Neunhoeffer F, et al. Newly developed Sr-substituted α-TCP bone cements. Acta Biomater. 2010;6(3):928–935. DOI:10.1016/j.actbio.2009.09.001 19733700

[52] Wu C, Zhou Y, Xu M, et al. Copper-containing mesoporous bioactive glass scaffolds with multifunctional properties of angiogenesis capacity, osteostimulation and antibacterial activity. Biomaterials. 2013;34(2):422–433. DOI:10.1016/j.biomaterials.2012.09.066 23083929

[53] Demura Y, Ameshima S, Ishizaki T, et al. The activation of eNOS by copper ion (Cu^2+^) in human pulmonary arterial endothelial cells (HPAEC). Free Radic Biol Med. 1998;25(3):314–320. DOI:10.1016/S0891-5849(98)00056-2 9680177

[54] Mayr-Wohlfart U, Waltenberger J, Hausser H, et al. Vascular endothelial growth factor stimulates chemotactic migration of primary human osteoblasts. Bone. 2002;30(3):472–477. DOI:10.1016/S8756-3282(01)00690-1 11882460

[55] Fiedler J, Leucht F, Waltenberger J, et al. VEGF-A and PlGF-1 stimulate chemotactic migration of human mesenchymal progenitor cells. Biochem Biophys Res Commun. 2005;334(2):561–568. DOI:10.1016/j.bbrc.2005.06.116 16005848

[56] Street J, Bao M, deGuzman L, et al. Vascular endothelial growth factor stimulates bone repair by promoting angiogenesis and bone turnover. Proc Natl Acad Sci U S A. 2002;99(15):9656–9661. DOI:10.1073/pnas.152324099 12118119 PMC124965

[57] Yang Q, McHugh KP, Patntirapong S, et al. VEGF enhancement of osteoclast survival and bone resorption involves VEGF receptor-2 signaling and β_3_-integrin. Matrix Biol. 2008;27(7):589–599. DOI:10.1016/j.matbio.2008.06.005 18640270

