# Supplementary material for: Angio-osteogenic capacity of octacalcium phosphate co-precipitated with copper gluconate in rat calvaria critical-sized defect
Source: Sci Technol Adv Mater. 2022 Feb 14;23(1):120–39. doi: 10.1080/14686996.2022.2035193 (PMC8856029; doi:10.1080/14686996.2022.2035193)
Supplement: Supplemental Material [file TSTA_A_2035193_SM7520.pdf]

Supplementary information

Angio-osteogenic capacity of octacalcium phosphate co-precipitated with copper gluconate in rat calvaria critical-sized defect

Shinki Koyama[a, b], Ryo Hamai[a], Yukari Shiwaku[a, c], Tsuyoshi Kurobane[a], Kaori Tsuchiya[a], Tetsu Takahashi [b], Osamu Suzuki[a]*

[a]*Division of Craniofacial Function Engineering, Tohoku University Graduate School of Dentistry, Sendai, Japan;* [b] *Division of Oral and Maxillofacial Surgery, Tohoku University Graduate School of Dentistry, Sendai 980-8575, Japan;* [c]*Liaison Center for Innovative Dentistry, Tohoku University Graduate School of Dentistry, Sendai, Japan*

*Corresponding author: Professor Osamu Suzuki, Ph.D., FBSE

Division of Craniofacial Function Engineering,

Tohoku University Graduate School of Dentistry,

4-1 Seiryo-machi, Aoba-ku, Sendai 980-8575, Japan

E-mail: suzuki-o@tohoku.ac.jp

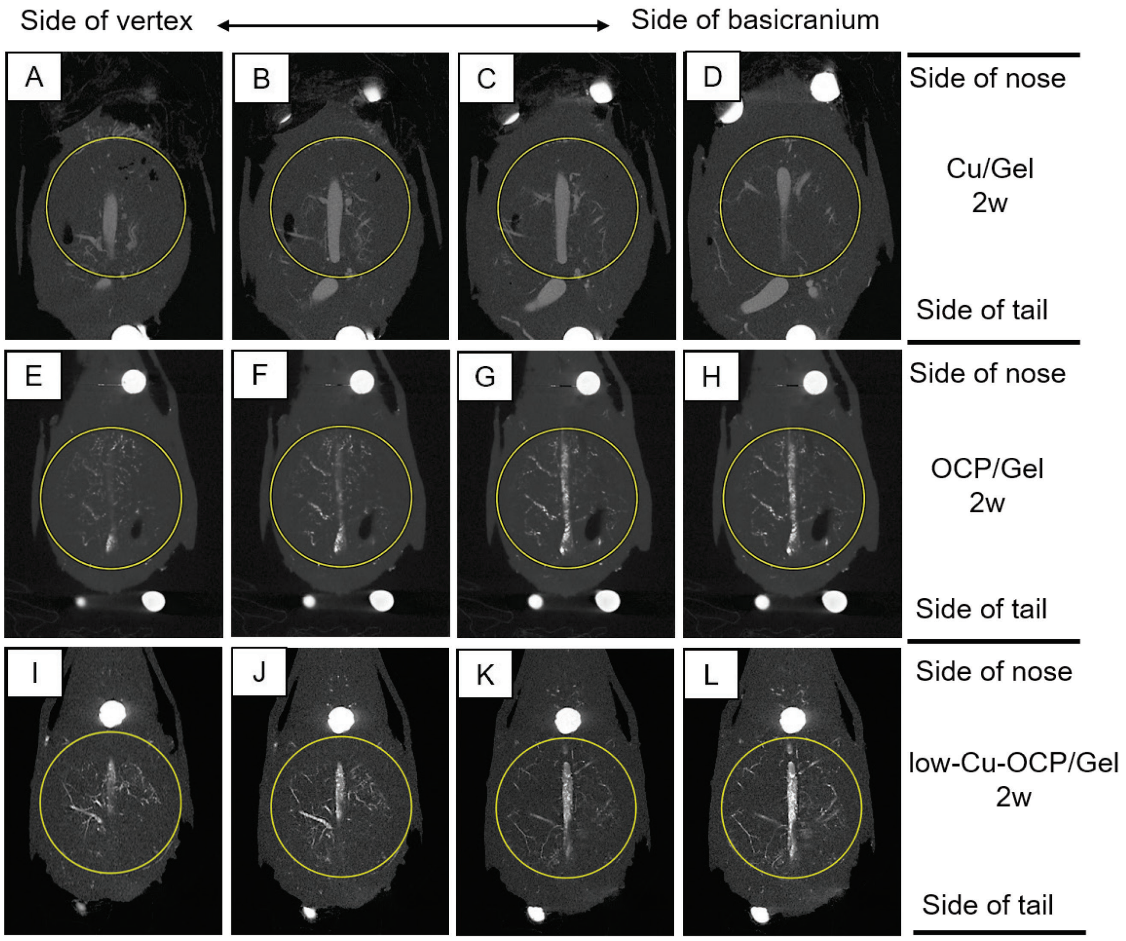

Figure S1. Cross-section in transverse plane of micro-CT images of rat calvaria around the defect after decalcification at 2 weeks post implantation. (A–D), Cu/Gel, (E–H) OCP/Gel, (I–L) OCP/Gel. Inside of yellow circles indicates region of defects. Inside of yellow circles indicates region of defects. Part of image was represented in Figure 8A in Main text.

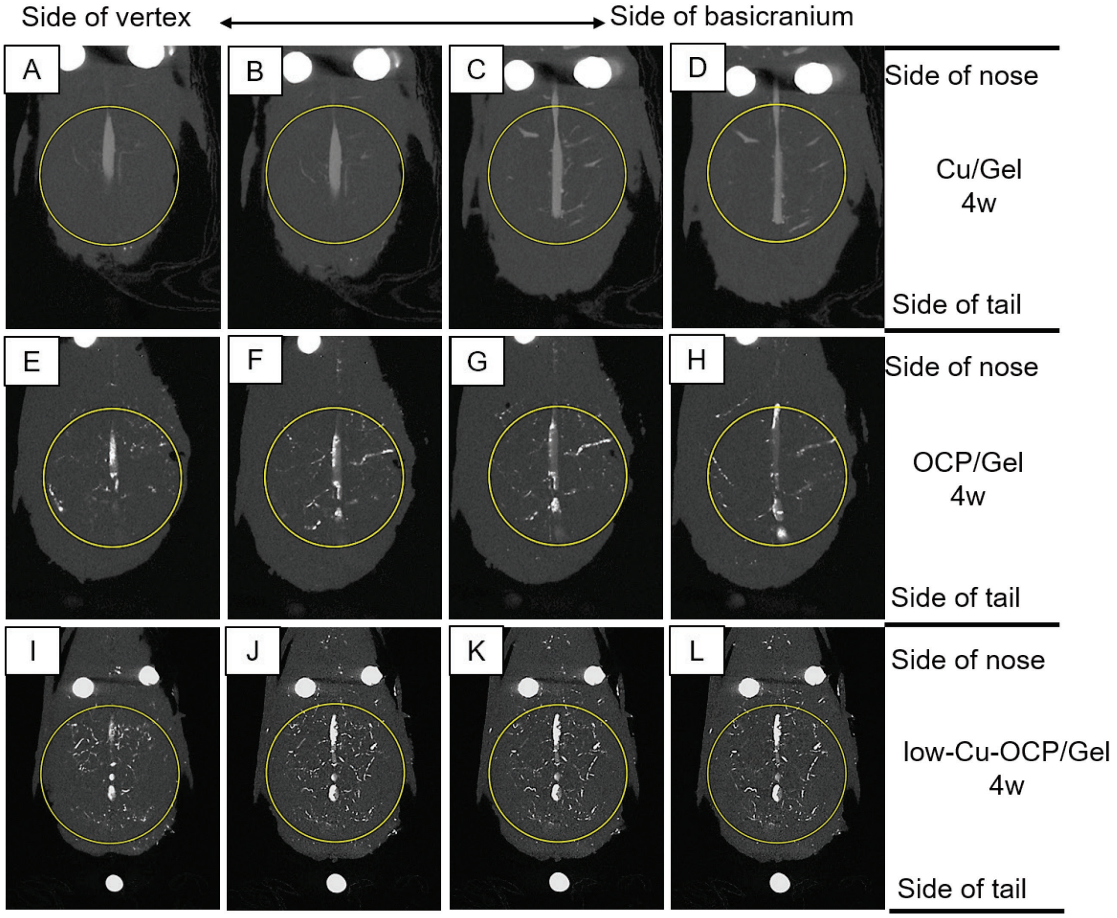

Figure S2. Cross-section in the transverse plane of micro-CT images of rat calvaria around the defect after decalcification at 4 weeks post implantation. (A–D), Cu/Gel, (E–H) OCP/Gel, and (I–L) OCP/Gel. Inside of yellow circles indicates the region of defects. Part of image was represented in Fig. 8A in Main text.

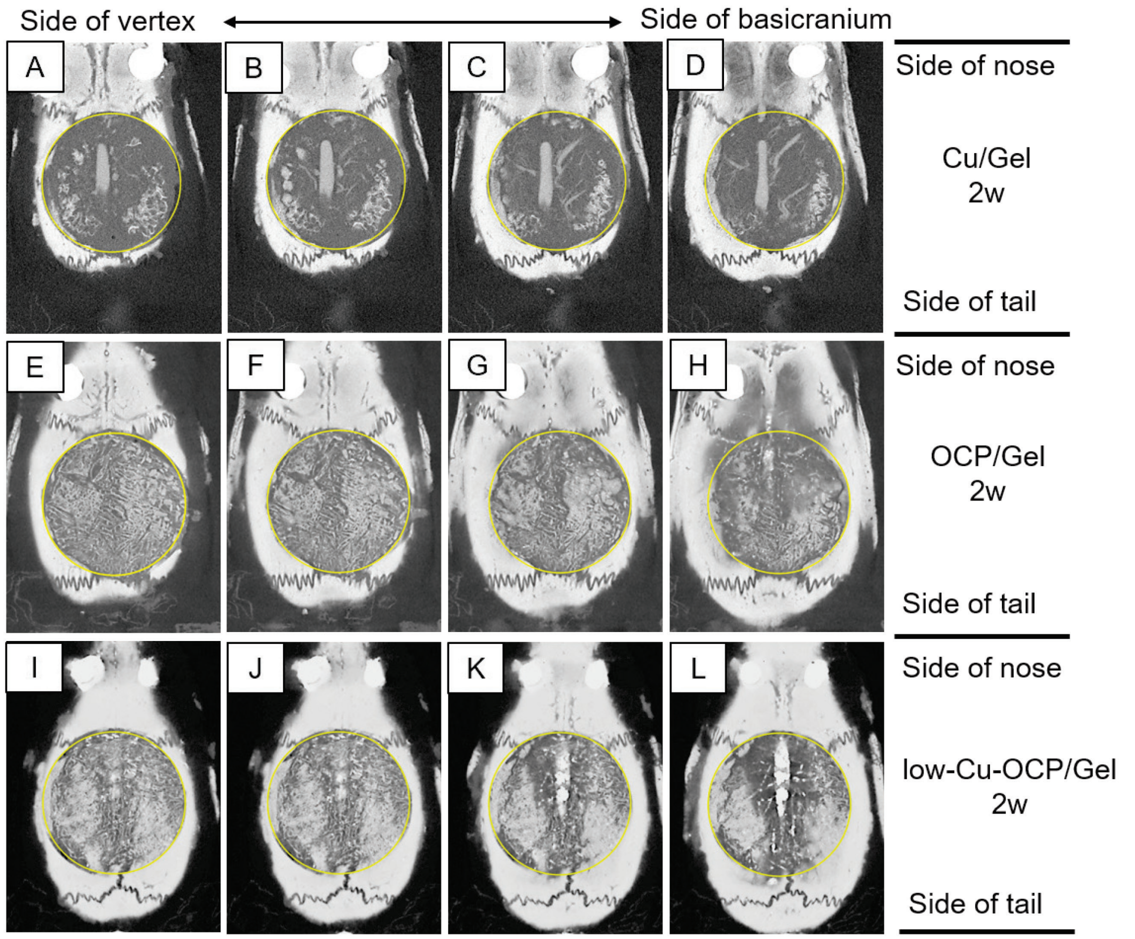

Figure S3. Cross-section in the transverse plane of micro-CT images of rat calvaria around the defect before decalcification at 2 weeks post implantation. (A–D), Cu/Gel, (E–H) OCP/Gel, and (I–L) OCP/Gel. Inside of yellow circles indicates the region of defects. Part of image was represented in Fig. 9A in Main text.

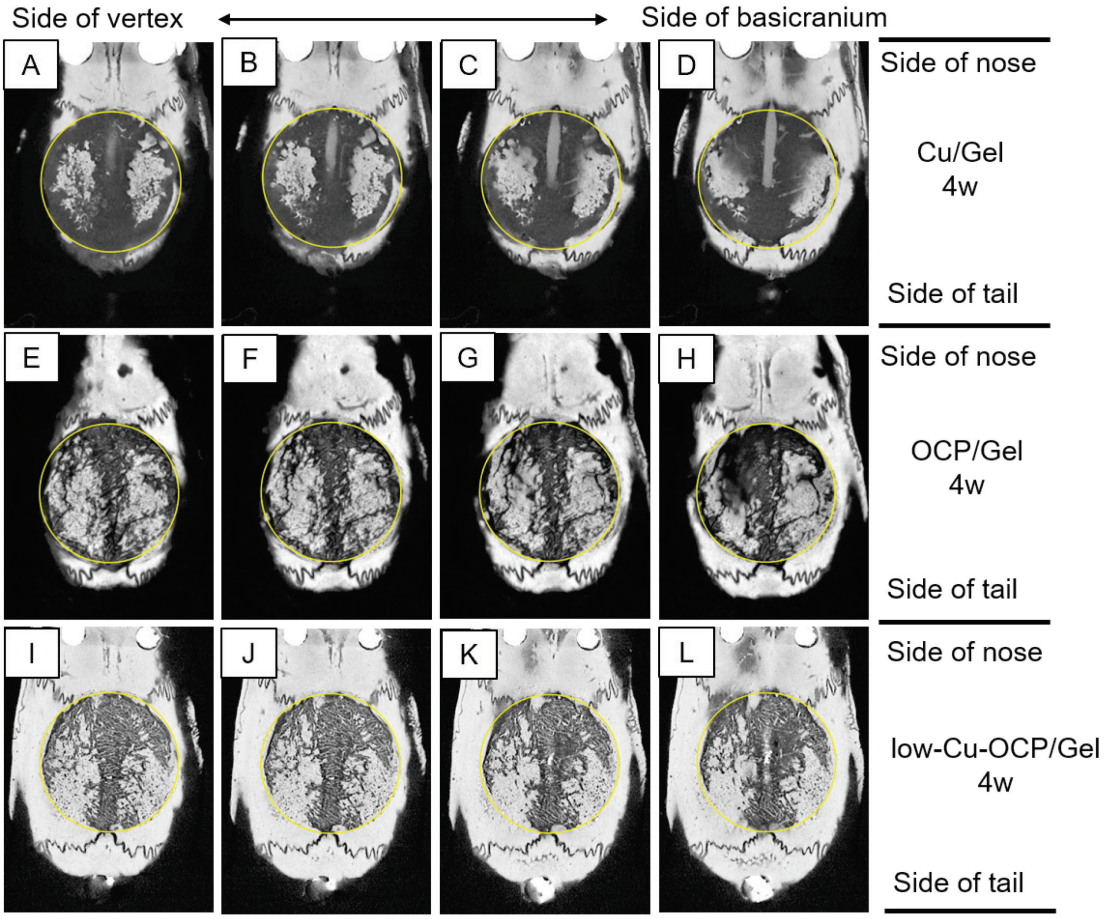

Figure S4. Cross-section in the transverse plane of micro-CT images of rat calvaria around the defect before decalcification at 4 weeks post implantation. (A–D), Cu/Gel, (E–H) OCP/Gel, and (I–L) OCP/Gel. Inside of yellow circles indicates the region of defects. Part of image was represented in Fig. 9A in Main text.
